# Differential Modes of Action of α1- and α1γ2-Autoantibodies Derived from Patients with GABA_A_R Encephalitis

**DOI:** 10.1523/ENEURO.0369-22.2022

**Published:** 2022-12-09

**Authors:** Adriana C. M. van Casteren, Frauke Ackermann, Kazi Atikur Rahman, Ewa Andrzejak, Christian Rosenmund, Jakob Kreye, Harald Prüss, Craig C. Garner, Aleksandra Ichkova

**Affiliations:** 1 Neurowissenschaftliches Forschungszentrum (NWFZ), Charité–Universitätsmedizin, Berlin 10117, Germany; 2 NeuroCure Cluster of Excellence, Charité–Universitätsmedizin, Berlin 10117, Germany; 3 German Center for Neurodegenerative Diseases (DZNE), Berlin 10117, Germany; 4 Einstein Center for Neurosciences Berlin, Charité- Universitätsmedizin, Berlin 10117, Germany; 5 Institute of Neurophysiology, Charité–Universitätsmedizin, Berlin 10117, Germany; 6 Department of Neurology and Experimental Neurology, Charité–Universitätsmedizin, Berlin 10117, Germany; 7 Department of Pediatric Neurology, Charité–Universitätsmedizin, Berlin 10117, Germany

**Keywords:** autoantibodies, autoimmune encephalitis, cortical/striatal neurons, GABAAR, network excitability

## Abstract

Autoantibodies against central nervous system proteins are increasingly being recognized in association with neurologic disorders. Although a growing number of neural autoantibodies have been identified, a causal link between specific autoantibodies and disease symptoms remains unclear, as most studies use patient-derived CSF-containing mixtures of autoantibodies. This raises questions concerning mechanism of action and which autoantibodies truly contribute to disease progression. To address this issue, monoclonal autoantibodies were isolated from a young girl with a range of neurologic symptoms, some of which reacted with specific GABA_A_ receptor (GABA_A_R) subunits, α1-subunit and α1γ2-subunit, which in this study we have characterized in detail using a combination of cellular imaging and electrophysiological techniques. These studies in neurons from wild-type mice (C57BL/6J; RRID:IMSR_JAX:000664) of mixed-sex revealed that the α1 and α1γ2 subunit-specific antibodies have differential effects on the GABA_A_ receptor. Namely, the α1-antibody was found to directly affect GABA_A_ receptor function on a short time scale that diminished GABA currents, leading to increased network excitability. On longer time scales those antibodies also triggered a redistribution of the GABA_A_ receptor away from synapses. In contrast, the α1γ2-antibody had no direct effect on GABA_A_ receptor function and could possibly mediate its effect through other actors of the immune system. Taken together, these data highlight the complexity underlying autoimmune disorders and show that antibodies can exert their effect through many mechanisms within the same disease.

## Significance Statement

It is increasingly apparent that neural autoimmune disorders can emerge as a consequence of immune responses to neural antigens. A precise diagnosis is further complicated, as individual patients express a combination of autoantibodies against several targets and/or to different epitopes on the same protein. It has therefore become critical to study the actions of individual autoantibodies to better understand their causal relationship and mode of action underlying patient’s symptoms. In characterizing the difference between two monoclonal antibodies isolated from a single patient, we show that their distinct modes of action can contribute to the complexity of such autoimmune disorders, highlighting difficulties in both diagnosis and antigen-specific therapies.

## Introduction

GABA_A_ receptor encephalitis (GABA_A_RE) is a disease characterized by the presence of autoantibodies targeting the GABA_A_ Receptor (GABA_A_R) and is associated with symptoms that include hallucinations, abnormal movement, epilepsy, and altered cognition, behavior, and consciousness (Ohkawa et al., 2014; Petit-Pedrol et al., 2014; Pettingill et al., 2015). Current treatment consists of a combination of corticosteroids and plasma exchange (O’Connor et al., 2019) and/or immunosuppressants (Lancaster, 2016; Shin et al., 2018). However, at present, the molecular and cellular mechanisms by which these antibodies cause disease progression are still rudimentary.

The predominant GABA_A_R autoantibody targets include ɑ1, β3, and γ2 subunits of the GABA_A_Rs (Ohkawa et al., 2014; Petit-Pedrol et al., 2014; Pettingill et al., 2015). GABA_A_Rs are ionotropic receptors involved in both phasic and tonic inhibition within neuronal circuits. They are assembled as pentamers with the most dominant receptor isoform being α1γ2β2α1β2 (Sigel and Steinmann, 2012; Ohkawa et al., 2014), which are expressed in a region and cell type-dependent manner (Mortensen et al., 2011). Subunit interfaces create binding sites not only for the neurotransmitter GABA but also for modulators such as benzodiazepine, which, for example, binds with high affinity to sites located between the α1, α2, α3, α5, and γ2 subunit combinations (e.g., α1γ2), increasing the affinity for GABA and receptor opening times (Sigel and Steinmann, 2012).

Initial studies on GABA_A_RE focused mainly on characterizing the long-term effects (>24 h) of patient-derived CSF *in vitro* within neuronal cultures. These studies revealed a loss of GABA_A_R clusters from inhibitory synapses following a 24-h or longer incubation with CSF (Ohkawa et al., 2014; Petit-Pedrol et al., 2014; Pettingill et al., 2015) as well as reduction in miniature IPSCs (mIPSCs; Ohkawa et al., 2014), indicating that such antibodies may directly increase neuronal network excitability and epilepsy by the loss of these receptors from synapses.

A major limitation to these and related studies is that patient CSF generally contains a mixture of several autoantibodies, only some of which are selective for GABA_A_Rs, raising questions about which are truly responsible for the observed symptoms. This has been largely resolved by isolating and cloning monoclonal antibodies from patient derived B-cells (Kreye et al., 2016) including from an eight-year-old girl with GABA_A_RE (Kreye et al., 2021), who presented at the hospital with fever, fatigue, reduced appetite, and apathy, which rapidly developed into severe confusion and mutism (Nikolaus et al., 2018). In the present study, we have performed a detailed cellular and molecular characterization of two of these antibodies, one that selectively recognized the α1 subunit and a second that recognized α1 and γ2 subunits (hereafter α1γ2) of the GABA_A_R. Their current mode of action is not well understood, but could involve receptor internalization (Petit-Pedrol et al., 2014; Pettingill et al., 2015; Jain and Balice-Gordon, 2016) or direct effects on GABA_A_Rs, depending on whether they bind an activation, neutral or modulatory site on these receptors (Diamond et al., 2009). This latter option is particularly attractive for the α1γ2-antibody which presumably binds the interface of these two subunits, perhaps affecting actions at the benzodiazepine site.

To address these issues, we have performed a detailed analysis of how the α1-antibody and α1γ2-antibody affect the distribution and function of GABA_A_Rs impact on neuronal network excitability as well as sensitivity to benzodiazepines in cultured striatal/cortical neurons. Our results show that the α1-antibody potently and rapidly affects spiking activity and GABA_A_R function (<5 min), independent of receptor cross-linking and internalization, and has a long-term effect (>20 h) on the distribution and function of synaptic GABA_A_Rs. In contrast, the α1γ2-antibody minimally effected the functionality of GABA_A_Rs, their sensitivity to benzodiazepine or striatal/cortical neuron excitability, suggesting its mode of action is indirect, perhaps via microglia-mediated immune response to this antibody. Together, these data indicate that autoantibodies can act through a variety of mechanisms to alter receptor and network function, contributing to the etiology of these diseases.

## Materials and Methods

### Neuronal culture preparation

All experiments with animal material were designed according to criteria set by the animal welfare committee of the Charité Medical University and the Berlin state government. Cells were isolated according to the “Banker Protocol” (Banker and Goslin, 1988; Meberg and Miller, 2003) from wild-type mice (C57BL/6J; RRID:IMSR_JAX:000664) of mixed-sex. In short, striatum and cortexes of postnatal day (P)0–P2 mice were dissected and incubated in enzyme solution (DMEM, Invitrogen, Thermo Fisher Scientific; 3.3 mm cysteine, 2 mm CaCl_2_, 1 mm EDTA, and 20 U/ml papain, Worthington) for 45 min at 37°C. Subsequently, inactivation solution (10% FCS, Thermo Fisher Scientific; 38 μm BSA, Sigma-Aldrich; and 178 μm trypsin inhibitor, Sigma-Aldrich; in DMEM) was added for 5 min to stop the digestion. Neurons were isolated by gentle trituration in NBA-complete (Neurobasal-A, Thermo Fisher Scientific; 2% B27, Invitrogen #17504; 1% glutamax, Invitrogen #35050; 0.2% penicillin/streptomycin, Roche #11074440001) and plated at different densities and in various preparations, specified below. Neuronal cells for each of these preparations were cultured for 14–18 d *in vitro* (DIV) in NBA-complete before subsequent use.

Autaptic striatal cultures were prepared by plating single striatal neurons onto astrocyte microislands on glass coverslips, prepared as described previously (Furshpan et al., 1976; Bekkers and Stevens, 1991). In short, acid-washed coverslips were covered in a thin layer of 0.15% agarose to discourage astrocytes and neurons from adhering. Then, small dots containing 0.25 mg/ml collagen and poly-D-lysine were added to the coverslip with the help of a stamp (Rost et al., 2015), to ensure that astrocytes and neurons would only adhere in these places. Later, astrocytes were isolated from P0–P2 wild-type mice cortices and plated at a density of 10,000 cell/cm^2^ for 7 d before the addition of neurons. Next, striatal neurons were plated at 500 cells/cm^2^ to ensure only one neuron per astrocyte island.

For both immunocytochemistry and electrophysiological experiments cortical-striatal mass-cultures were prepared. Here, astrocytes were plated at a density of 18,500 cells/cm^2^ directly into cell-culture plates and grown for 7 d. Striatal and cortical neurons, prepared on day 7 were plated at a ratio of 3:1 and at a density of 60,000 cells/cm^2^ on glass coverslips that were acid washed and poly-L-lysine coated previously (Tian et al., 2010; Penrod et al., 2011; Lalchandani et al., 2013). These coverslips were decorated with paraffin dots and were then placed upside down over the astrocyte covered cell-culture plates.

For calcium imaging experiments, astrocytes were also plated 7 d before the direct addition of neurons at a density of 18,500 cells/cm^2^. Here, cells were plated directly into round dishes containing a cell location grid at the bottom (μ-Dish 35 mm, high Grid-500, Ibidi), allowing the repetitive imaging of the same cells. For some calcium imaging experiments, both astrocytes and neurons were directly plated on PDL-coated coverslip. In both cases, striatal and cortical neurons were plated in a ratio of 3:1 at a high density of 120,000 cells/cm^2^ directly on top of the astrocyte feeder layer. In each case, after 7 DIV, one-third of the culture media was replaced with fresh NBA-complete, which was repeated every 2–3 d until the experimental day.

### Monoclonal antibodies

The α1-antibody for the 113–115 clone and α1γ2-antibody for the 113–175 clone described previously (Kreye et al., 2021) were kindly provided by Harald Prüß (Charité–Universitätsmedizin, Berlin).

### Electrophysiological recordings

We performed whole-cell patch-clamp recordings in two different neuronal-culture preparations. First, we recorded from striatal autaptic cultures that were incubated with 1 μg/ml of α1-antibody, α1γ2-antibody, or control-antibody (human anti-alemtuzumab, Bio-Rad catalog #HCA175, RRID:AB_11152938) for 24 h before the onset of the experiment. Second, we also recorded neurons in cortical-striatal mass-cultures, where neurons were treated 1 h before experiment onset with 1 μg/ml of control or the α1-antibody, or 5 μg/ml of the α1γ2-antibody. In general, recordings were performed at room temperature under the control of Clampex 10.4 software (Molecular Devices), and neurons were voltage clamped at −70 mV with a Multiclamp 700B amplifier (Molecular Devices, RRID:SCR_018455). Data were sampled at 10 kHz and filtered through a 3-kHz Bessel filter. Series resistance was kept under 15 MΩ and were compensated to ∼70%. During all recordings, the leak current was monitored and any recordings with a leak larger than −300 pA were discarded. All cells were kept in extracellular solution during recordings, which consisted of 140 mm NaCl, 2.4 mm KCl, 10 mm HEPES, 10 mm glucose, 2 mm CaCl_2_, and 4 mm MgCl_2_. Whole-cell patch-clamp recordings were obtained through borosilicate glass pipettes (3–8 MΩ) pulled with a micropipette puller device (Sutter Instruments). These pipettes were filled with internal solution containing 136 mm KCl, 17.8 mm HEPES, 1 mm EGTA, 0.6 mm MgCl_2_, 4 mm ATP-Mg, 0.3 mm GTP-Na, 12 mm phosphocreatine, and 50 U/ml phosphocreatine kinase. All solutions were adjusted to pH 7.4 and osmolarity of ∼300 mOsm.

For 24-h striatal autapse experiments, all recordings were performed in standard intracellular and extracellular solutions, except when bath-applying NMDA, which was measured in extracellular solution containing 0 mm Mg2+, 0.2 mm CaCl_2_, and 10 μm glycine. First, we evoked IPSCs through a 2-ms somatic depolarization from −70 to 0 mV to probe the synaptic GABA_A_R response. Subsequently, IPSCs were evoked in the presence of bicuculline (30 μm, Santa Cruz #sc-202498) to assess whether cells were indeed inhibitory neurons. From traces in control solution, miniature IPSCs (mIPSCs) were recorded and analyzed after 1-kHz low-pass filtering using a templated based detection feature Axograph X (RRID:SCR_014284). False negative events were excluded by substracting events detected in GABA_A_ receptor antagonist bicuculline. To quantify the synaptic and extrasynaptic surface glutamate and GABA_A_ receptor activity, responses to pulsed application of kainate (1 s, 20 μm, Tocris #0222), NMDA (1 s, 10 μm, Tocris #0114), and GABA (3 s, 5 μm, Tocris #0344) were recorded. The size of the readily releasable pool of vesicles pool was determined by applying extracellular solution that contained additionally 0.5 mm sucrose (Rosenmund and Stevens, 1996).

When recording from cortical-striatal mass-cultures, mIPSCs were recorded in standard extracellular solution plus tetrodotoxin (TTX; 0.5 μm, Tocris #1078), AP5 (50 μm, Tocris #0106), and NBQX (10 μm, Tocris #1044). The mIPSCs were recorded for a 1-min period after 1 h of antibody incubation, followed by a 1-min recording in the presence of diazepam (1 μm, Sigma-Aldrich #D0899) and bicuculline (30 μm, Santa Cruz #sc-202498). Here also, bicuculline traces were used for noise correction.

All electrophysiological data were analyzed with Axograph X, Excel (Microsoft), and Prism (GraphPad).

### Immunocytochemistry

For immunocytochemistry experiments, primary cortical-striatal co-cultures were treated with 1 μg/ml of α1-antibody or α1γ2-antibody. Antibodies were incubated at 37°C for 24 h or at 15°C for 1 h. After treatment, cells were fixed for 4 min with 4% PFA in PBS and subsequently quenched for 20 min with 25 mm glycine in PBS. All following steps were performed in blocking serum (2% BSA, and 5% normal goat serum in PBS) unless otherwise stated. First, cells were blocked and permeabilized in 0.2% triton for 1 h. Afterwards, cells were incubated for 1 h with secondary Alexafluor-594-anti-human antibodies (1:1000, Jackson #109-585-003). Next, cells were washed three times 5 min in blocking serum, after which primary antibodies, chicken-anti-MAP2 (1:2000, Millipore catalog #AB5543, RRID:AB_571049) and rabbit-anti-VGAT (1:2000, Synaptic Systems catalog #131003, RRID:AB_887869), were added for 1 h. Again, cells were washed three times with blocking serum before adding secondary antibodies for 1 h at a 1:1000 concentration: e.g., Alexafluor-405-anti-chicken (Abcam catalog #ab175674, RRID:AB_2890171) or Alexafluor-488-anti-chicken (Thermo Fisher Scientific catalog #A-11 039, RRID:AB_2534096) and Alexafluor-647-anti-rabbit (Thermo Fisher Scientific catalog #A-21 245, RRID:AB_2535813). Finally, cells were washed one more time with blocking serum and two times in PBS before mounting coverslips with Mowiol (10 mm Mowiol 4-88, Roth #0713.2; 3.6 m glycerol; 0.2 m Tris in distilled water, pH 8.5).

### Image acquisition and quantification

All immunocytochemically stained neurons were imaged on a spinning disk confocal microscope (Zeiss Axio Observer.Z1 with an Andor spinning disk and cobolt, omricron, i-beam laser) using a 40× (1.3 NA) oil objective and an iXon ultra (Andor) camera controlled by iQ software (Andor). Immunocytochemistry of both time points, 24 and 1 h, was performed at the same time and imaged with the same setting across all conditions. Per condition, 10 neurons were selected based on the MAP2 signal and Z-stack (Z = 4) images were taken. Images were processed using ImageJ (RRID:SCR_003070). In brief, maximum projections were created from each Z-stack after which three dendrites per neuron were selected via the MAP2 channel and analyzed with a custom-written script for ImageJ analysis. The script subtracts the background with a rolling stack of 3. Next, it opens Trackmate, a standard ImageJ plugin, wherein blob diameter is set to three and threshold is manually selected to include all puncta in either the GABA_A_R antibody channel or the VGAT channel. The mean fluorescent values of all detected puncta are then exported to excel file (Microsoft) and analyzed to see whether VGAT and autoantibody spots overlap. Puncta were considered “positive” for a given marker when the mean fluorescent value (au) was bigger than 2× the background signal. Per experiment, three cultures were stained. Normalized means of intensity in puncta represent the means of all puncta normalized to the in-culture 1-h average intensity, this was done to account for variations in staining intensity between cultures.

### Virus production

For calcium imaging experiments, the Viral Core Facility of the Charité Medical University Berlin (https://vcf.charite.de/en/) produced a f(syn)-NES-jRCamP1b-WPRE-w lentivirus. Production occurred as described previously (Lois et al., 2002).

### Calcium imaging experiments

To visualize calcium influx, cortical-striatal primary co-cultures were infected on DIV 4 with a f(syn)-NES-jRCamP1b-WPRE-w lentivirus which expresses jRCamP1b under the Synapsin promoter. From DIV 7 on, one-third of the media was replaced with fresh NBA-complete every 2–3 d until experiment onset (DIV 14–18). Neurons were imaged using a Nikon Spinning Disk Confocal CSU-X microscope with a 20× Plan Apo air-objective (NA = 0.8), controlled via the NIS-Elements software (Nikon) in the Charité AMBIO facility. For each imaging session, cells were kept at 37°C and 5% CO_2_ during image acquisition and were imaged with a 561 laser, an exposure time of 50 ms, and 300 gain, at 0.2 Hz for 2 min.

To assess long-term effects, auto-antibodies were added 24 h before the onset of imaging at selected concentrations (1 μg/ml for α1-antibody, α1γ2-antibody, or control-antibody, and 5 μg/ml of α1γ2-antibody). For each experiment, three coverslips per condition were selected with three regions of interest (ROIs) per coverslip. For ROIs, we selected areas that had 10–15 neurons that showed spontaneous activity. During the imaging session, all three ROIs were imaged in succession after which all three ROIs were immediately imaged for a second time. During analysis, the averages spike activity of the two recordings was averaged per ROI to ensure a stable activity count over time. Afterward, bicuculline (30 μm, Santa Cruz #sc-202498) was added to the cultures, and after a 1-min waiting period to allow diffusion, the same ROIs were imaged for two consecutive times.

For 1-h and benzodiazepine experiments, antibodies were added 1 h before the imaging session. Subsequently, the three ROIs were imaged twice without any drugs for the 1-h experiments, then twice with diazepam (1 μm, Sigma-Aldrich #D0899) for the benzodiazepine experiments, and finally twice with bicuculline (30 μm, Santa Cruz #sc-202498). For these experiments, cells were initially imaged in 500 μl NBA-complete (2% B27 Invitrogen #17504, 1% glutamax Invitrogen #35050, 0.2% penicillin/streptomycin Roche #11074440001). Afterwards, 500 μl NBA-complete with a 2× Diazepam concentration was added. After Diazepam imaging, 900 μl was removed from the imaging chamber and 900-μl NBA-complete with bicuculline was added.

For acute antibody addition, only one ROI per coverslip was imaged. This ROI was imaged for 2 min in 500-μl NBA-complete solution, after which 500 μl of NBA-complete with 2 μg/ml of the α1-antibody or 4 μg/ml of the α1-fab-antibody was added, resulting in a 1 or 2 μg/ml final solution, respectively. After the addition of the antibody, the same ROI was imaged for an additional 12 min, to assess its effect on neuronal spiking activity.

After data acquisition, images were analyzed with the help of OpenView software (Noam Ziv, Technion Institute, Haifa, Israel) and a script written by Noam Ziv in Excel (Microsoft). In brief, ROIs were manually selected by placing boxes of 27 × 27 pixels over 10–15 visually identified neuronal cell somas. Only active cells were included in the analysis. To detect somatic calcium transients, time-series fluorescence values were converted into ΔF/F by calculating the ratio between the change in fluorescence signal intensity (δ F) and baseline fluorescence (F0). The custom-written algorithm identified the timestamps of calcium transient onset, which were then averaged per minute to obtain the frequency of events.

### Code accessibility

For the analysis of calcium imaging experiments, we used custom-written software (“OpenView”; available on request; analyses could also be performed using other software packages, such as ImageJ) written by Noam Ziv (Technion Institute, Haifa, Israel). For access to the code please kindly contact Noam Ziv.

### Experimental design and statistical analyses

In each figure legend, details about the experimental design are mentioned. Each experiment represents three independent cultures. All statistical tests and graphs were represented with the help of GraphPad Prism (RRID:SCR_002798). Information on statistical tests and sample sizes can be found in each figure legend. In general, when comparing two groups, we used paired and unpaired *t* tests, and in the case of unequal variances, a Welch’s correction was applied. When there were more than two conditions, we compared the means via ordinary one-way or repeated measures ANOVA followed by Tukey’s multiple comparisons test, unless either the Brown–Forsythe or Barlett’s test showed unequal variances. In that case, significance was tested with a Kruskal–Wallis test followed by Dunn’s multiple comparisons. For acute calcium imaging experiment, we used repeated measures two-way ANOVA with Sidak’s multiple comparisons. Means are reported with SEM. For the sake of readability, we mention only mean ± SEM and *p*-values in the text, test statistics and *post hoc* analysis can be found in the figure legends.

## Results

### Long-term effects of α1-antibody and α1γ2-antibody on GABA_A_ receptor distribution

Based on previous studies with patient derived CSF, it is expected that autoantibodies targeting GABA_A_Rs will affect receptor distribution. To explore whether the α1- and α1γ2-monoclonal-antibodies also have a long-term effect on GABA_A_ receptor distribution, we incubated primary cortico-striatal cultures with each of these antibodies for 1 or 24 h, before processing for immunocytochemistry. The intrinsic surface binding of each antibody was initially defined by incubating cultures for 1 h at 15°C with 1 μg/ml of either antibody, while their effects on the redistribution of these receptors was accomplished by incubating cultures with antibody for 24 h at 37°C. Of note, the longer time point was used to mirror the *in vivo* situation, as performed previously (Moscato et al., 2014; Ohkawa et al., 2014; Petit-Pedrol et al., 2014). Cultures were then fixed and immune-stained with fluorescent secondary antibodies as well as antibodies against the vesicular GABA transporter VGAT (GABA synapse marker) and MAP2 (a dendrite-specific microtubule-associated protein).

**Figure 1.**
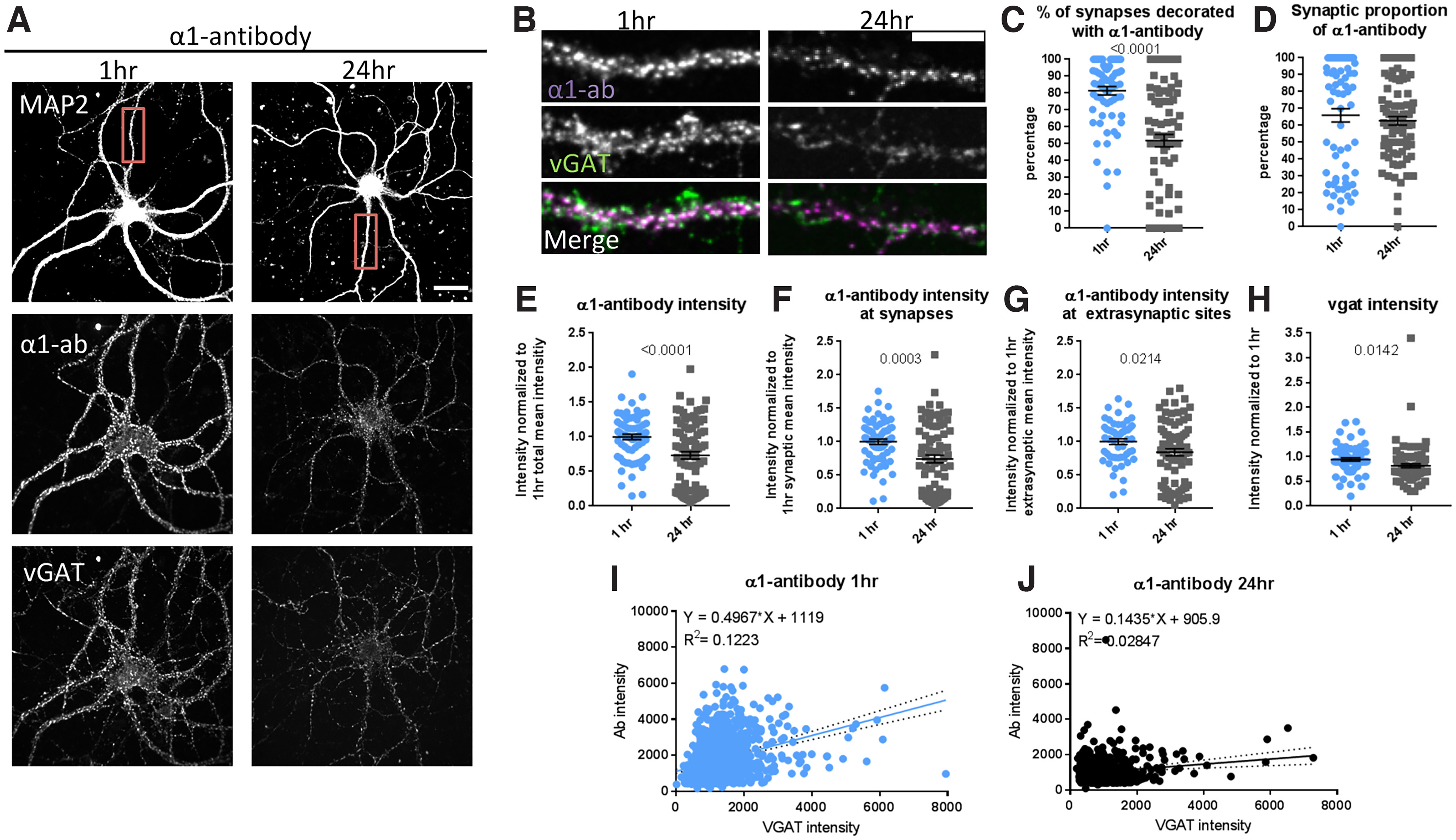
α1-antibody affects receptor distribution over time. ***A***, Staining of cortical-striatal cultures (1:3) with MAP2 (white), VGAT (green), and α1-antibody (red) after 1-h α1-antibody incubation at 15°C and for 24 h at 37°C; scale bar: 20 μm. ***B***, Close-up of boxed area in ***A***, showing synaptic colocalization between VGAT and α1-antibody; scale bar: 10 μm. ***C***, Over time, the α1-antibody leads to reductions in percentage of synapses covered with α1-antibody puncta, *t*_(146.4)_ = 6.664, *p* < 0.0001, Welch’s *t* test, while (***D***) the α1-antibody can be found equally at synaptic and extrasynaptic sites over time, *t*_(121)_ = 0.691, *p* = 0.4907, Welch’s *t* test. In addition, (***E***) long antibody incubation (24 h) leads to reductions in overall α1-antibody intensity, *t*_(148.5)_ = 4.11, *p* < 0.0001, Welch’s *t* test, (***F***) because of reduction in intensity at synaptic sites, *t*_(136.4)_ = 3.701, *p* = 0.0003, Welch’s *t* test, (***G***) and extrasynaptic sites *t*_(129.8)_ = 2.329, *p* = 0.0214, Welch’s *t* test. ***H***, Overall, VGAT intensity is also decreased over time, *t*_(156.1)_ = 2.479, *p* = 0.0142, Welch’s *t* test. This is also seen in histograms when VGAT intensity is plotted against the α1-antibody intensity at 1 h (***I***) versus at 24 h (***J***), where we observed a reduction in the slope of the correlation because of a decrease in α1-antibody intensity. Each data point represents one ROI, except in ***I*** and ***J***, where each data point represents a synapse. Error bars represent SEM.

Comparing the staining pattern of the α1-antibody against VGAT, we observed that this antibody decorated all neurons in a punctate pattern (Fig. 1*A*,*B*) labeling around 81 ± 2% of all VGAT-positive inhibitory synapses at the 1-h time point (Fig. 1*C*). Over time (24 h), this high degree of co-localization decreased to 51 ± 4% (*p* < 0.0001, Welch’s corrected *t* test) indicating that this antibody promoted the exit of these receptors from synapses, perhaps by lateral diffusion or internalization (Fig. 1*A–C*). A more detailed analysis of the α1-antibody distribution revealed that, at 1 h, 66 ± 4% of all α1-antibody positive puncta were located at inhibitory synapses (Fig. 1*D*), while 34% were present at extrasynaptic sites. After 24 h, the synaptic fraction of α1-antibody puncta remained relatively the same with 63 ± 3% of the puncta located at synaptic sites (Fig. 1*D*). Moreover, the intensity of α1-antibody puncta decreases over time by ∼25% (*p* < 0.0001, Welch’s *t* test; 1 h, 1 ± 0.04, 24 h, 0.73 ± 0.05; Fig. 1*E*), which is because of reductions in intensity at synaptic sites (*p* = 0.0003, Welch’s *t* test; 1 h, 1 ± 0.04, 24 h, 0.74 ± 0.06; Fig. 1*F*), and at extrasynaptic sites (*p* = 0.0214, Welch’s *t* test, 1 h, 1 ± 0.04, 24 h, 0.84 ± 0.05; Fig. 1*G*). In addition, when the α1-antibody intensity is plotted against the VGAT intensity, we observe a reduction in the slope of the correlation (Fig. 1*I*,*J*). Intriguingly, this is associated with a decrease in VGAT puncta intensity of ∼10% (*p* = 0.0142, Welch’s *t* test; 1 h, 1 ± 0.03, 24 h, 0.82 ± 0.04), indicating that the antibody indirectly reduces the size of the presynaptic pool of synaptic vesicles (Fig. 1*H*). Together, these data indicate that the α1-antibody can exert part of its effect by triggering a redistribution of these receptors.

**Figure 2.**
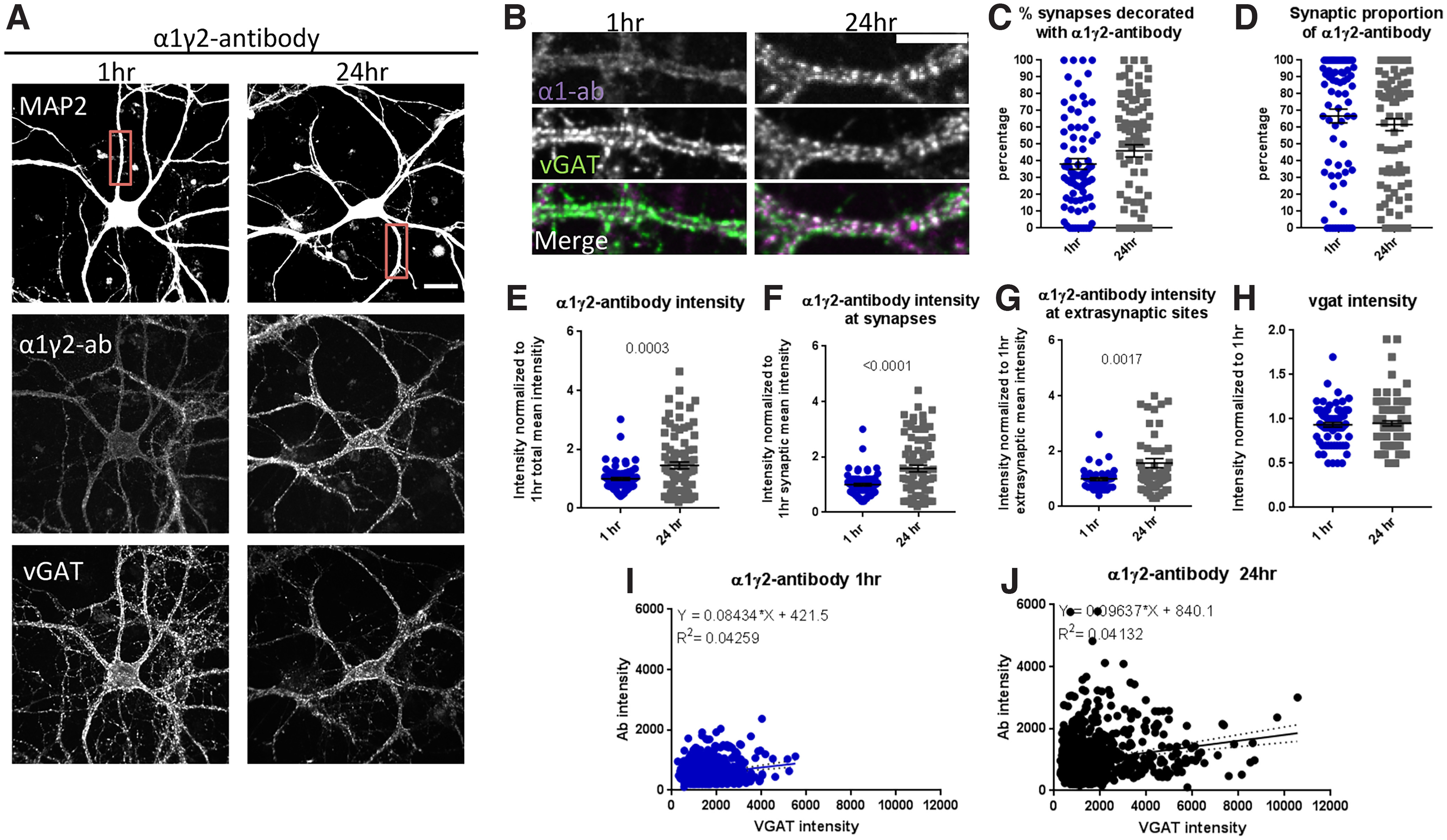
α1γ2-antibody stains neurons in a GABA_A_R-specific pattern. ***A***, Staining of cortical-striatal cultures (1:3) with MAP2 (white), VGAT (green), and α1γ2-antibody (red) after 1-h α1γ2-antibody incubation at 15°C or for 24 h at 37°C; scale bar: 20 μm. ***B***, Close-up of boxed area, showing synaptic colocalization between VGAT and α1γ2-antibody positive puncta; scale bar: 10 μm. ***C***, After incubation with the α1γ2-antibody, we observed that the percentage of VGAT puncta colocalizing with α1γ2-antibody puncta remain similar from 1 to 24 h, *t*_(163)_ = 1.62, *p* = 0.1072, unpaired *t* test, (***D***) as do the percentage of α1-antibody puncta that colocalize with VGAT puncta over time, *t*_(161)_ = 0.9327 *p* = 0.3524, unpaired *t* test. ***E***, Over time (24 h), the α1γ2-antibody addition is associated with increases in α1γ2-antibody puncta intensity, *t*_(109.8)_ = 3.702, *p* = 0.0003, Welch’s *t* test. When we split α1γ2-puncta intensity based on colocalization with VGAT puncta, we see an increase in both synaptic (***F***), *t*_(100.7)_ = 4.442, *p* < 0.0001, Welch’s *t* test, and extrasynaptic α1γ2-antibody intensity (***G***), *t*_(81.46)_ = 3.243, *p* = 0.0017, Welch’s *t* test. ***H***, However, the α1γ2-antibody does not alter VGAT intensity *t*_(158.4)_ = 0.4497, *p* = 0.6535, Welch’s *t* test. In histograms comparing VGAT versus α1γ2-antibody puncta intensity at 1 h (***I***) versus at 24 h (***J***), we observe no difference in the slope of the correlation, although auto-antibody intensity increases. Each data point represents one ROI, except in ***I*** and ***J***, where each data point represents a synapse. Error bars represent SEM.

Interestingly, based on colocalization experiments with VGAT, only ∼38 ± 3% of the VGAT positive puncta colocalized with the α1γ2-positive puncta following a 1-h incubation of primary cortico-striatal cultures with the α1γ2-antibody, after 24 h, this percentage was 46 ± 4% (Fig. 2*A–C*). Together, these data indicate that only a small percentage of the synapses contain this combination of subunits. When we looked at the synaptic and extrasynaptic distribution of α1γ2-positive puncta, we observed that 67 ± 4% of the α1γ2-antibody puncta were located at synapses, which was unaffected by a longer 24-h incubation (Fig. 2*D*). Intriguingly, the fluorescent intensity of α1γ2 puncta increased over time (1 h, 1 ± 0.40, 24 h, 1.45 ± 0.12, *p* = 0.0003, Welch’s *t* test; Fig. 2*E*) at both synaptic (1 h, 1 ± 0.05, 24 h, 1.58 ± 0.12, *p* < 0.0001, Welch’s *t* test; Fig. 2*F*) and extrasynaptic locations (1 h, 1 ± 0.05, 24 h, 1.57 ± 0.17, *p* = 0.0017, Welch’s *t* test; Fig. 2*G*). It is currently unclear why this occurs. It could be because of the antibody avidity, or it could trigger the clustering of receptors at, for example, inhibitory synapses. Of note, the VGAT puncta intensity was not affected by this antibody (Fig. 2*H*). In addition, the correlation between VGAT intensity and α1γ2-antibody intensity does not seem to change over time (Fig. 2*I*,*J*).

### α1-antibody but not the α1γ2-antibody reduces GABAergic currents after a 24-h treatment

**Figure 3.**
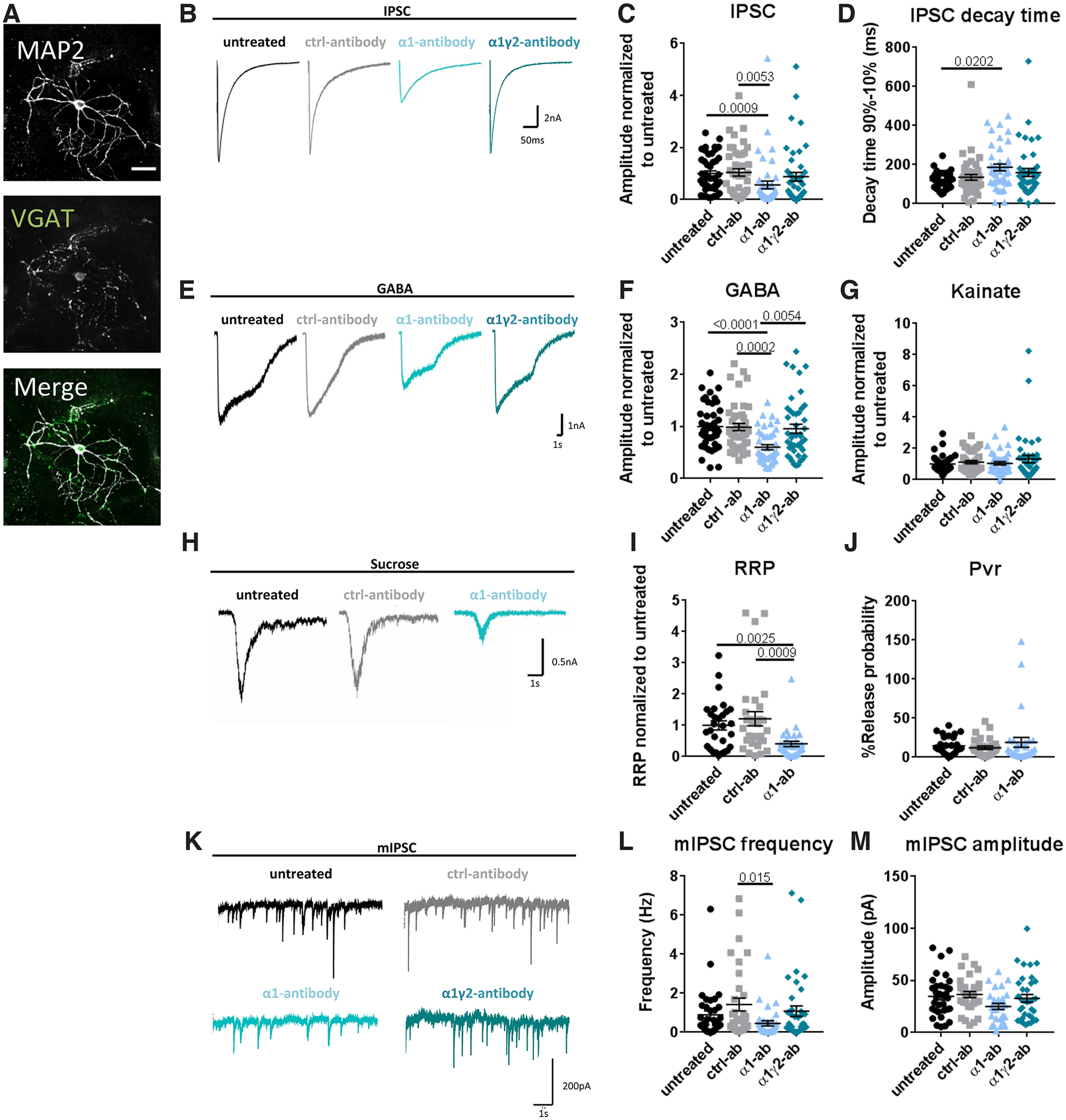
α1-antibody but not the α1γ2-antinbody reduces GABAergic currents after a 24-h treatment. ***A***, Staining of striatal autaptic neurons with MAP2 (white) and VGAT (green); scale bar: 20 μm. ***B***, Example IPSC traces for each condition. ***C***, Quantification of IPSC amplitude shows that autaptic neurons treated with α1-antibody for 24 h have reduced synaptic IPSC amplitudes, *H*_(3)_ = 17.78, *p* = 0.0005, Kruskal–Wallis; untreated 1 ± 0.10 versus α1-antibody 0.56 ± 0.15, *p* = 0.0009, control 1.04 ± 0.14 versus α1-antibody *p* = 0.0053, (***D***) and longer decay times, *H*_(3)_ = 9.79, *p* = 0.0205, Kruskal–Wallis; untreated 118.4 ± 5.93 ms versus α1-antibody 186.3 ± 17.68 ms, *p* = 0.0202. ***E***, Example traces current induced after bath application of 5 μm GABA show (***F***) that the presence of α1-antibody leads to reductions in GABA currents *H*_(3)_ = 25.28, *p* < 0.0001, Kruskal–Wallis; untreated 1 ± 0.06 versus α1-antibody 0.6 ± 0.05 *p* < 0.0001, control 0.99 ± 0.07 versus α1-antiody *p* = 0.0002, α1-antibody versus α1γ2-antibody 0.96 ± 0.09 *p* = 0.0054. ***G***, It does not affect excitatory currents induced by bath application of Kainate, *H*_(3)_ = 0.52, *p* = 0.9135, Kruskal–Wallis. ***H***, Representative traces of currents induced following the addition of sucrose show (***I***) that the ready releasable pool (RRP) is decreased in the presence of the α1-antibody, *H*_(3)_ = 16.2, *p* = 0.0003, Kruskal–Wallis; untreated 1 ± 0.15 versus α1-antibody 0.40 ± 0.08 *p* = 0.0025, control 1.21 ± 0.22 versus α1-antibody *p* = 0.0009, (***J***) but that release probability (Pvr) is not affected, *H*_(3)_ = 1.59, *p* = 0.4506, Kruskal–Wallis. Example traces of mIPSCs (***K***) show that (***L***) frequency was reduced after α1-antibody incubation compared with control-antibody conditions, *H*_(3)_ = 10.49, *p* = 0.0149, Kruskal–Wallis; control 1.42 ± 0.32 Hz versus α1-antibody 0.46 ± 0.14 Hz *p* = 0.0151, (***M***) but mIPSC amplitude remained the same, *H*_(3)_ = 6.94, *p* = 0.0737, Kruskal–Wallis. Incubation with the α1γ2-antibody (***K–M***) does not lead to any differences in synaptic currents. Each data point represents one cell. Error bars represent SEM.

A fundamental question is whether the observed changes in receptor redistribution also led to changes in GABA_A_R-mediated currents. This was initially investigated by performing whole-cell patch recording of inhibitory striatal neurons incubated for 24 h with each antibody. To look specifically at the effect of the antibody on total and synaptic GABA_A_R-mediated currents, we used striatal autaptic cultures, in which a single inhibitory striatal neuron is cultured on a microisland of astrocytes (Furshpan et al., 1976; Bekkers and Stevens, 1991; Fig. 3*A*), causing neurons to make synapses of a single type onto themselves.

For autaptic striatal neurons incubated with the α1-antibody for 24 h, we observed an ∼45% reduction in evoked synaptic GABA currents (untreated 1 ± 0.10, α1-antibody 0.56 ± 0.15, and control-antibody 1.04 ± 0.14, *p* = 0.0005, Kruskal–Wallis; Fig. 3*B*,*C*) with significantly longer decay times (untreated 118 ± 6 ms, control-antibody 135 ± 15 ms, and α1-antibody 186 ± 18 ms, *p* = 0.0205, Kruskal–Wallis; Fig. 3*D*). When 5 μm GABA was bath applied for 3 s, activating both synaptic and extrasynaptic GABA receptors, GABA currents were reduced by ∼40% after α1-antibody preincubation (*p* < 0.0001, Kruskal–Wallis; untreated 1 ± 0.06, α1-antibody 0.6 ± 0.05, control-antibody 0.99 ± 0.07, and α1γ2-antibody 0.96 ± 0.09 *p* = 0.0054; Fig. 3*E*,*F*), whereas bath application of 20 μm kainate (Fig. 3*G*) or 10 μm NMDA (data not shown, *p* = 0.9990, Kruskal–Wallis; untreated 1 ± 0.07, control-antibody 1 ± 0.09, α1-antibody 0.98 ± 0.07, α1γ2-antibody 1.16 ± 0.14) did not significantly change their associated currents. To tease apart whether the observed reduction in GABA currents was because of fewer postsynaptic receptors or because of less presynaptic GABA release, we investigated the sucrose responses, which directly trigger the release of docked synaptic vesicles (Stevens and Tsujimoto, 1995) and thus only activates synaptic receptors. The sucrose response revealed that the addition of the α1-antibody was associated with a smaller charge (*p* = 0.0003, Kruskal–Wallis; untreated 1 ± 0.15, α1-antibody 0.40 ± 0.08, and control-antibody 1.21 ± 0.22; Fig. 3*H*,*I*), however with no effect on release probability (Fig. 3*J*). These data indicate that the antibody induced changes in GABA currents are most likely because of postsynaptic alterations, e.g., change in number of functional postsynaptic GABA_A_Rs and/or total number of synapses. To further explore these options, we investigated characteristics of mIPSCs, which monitors the response of individual postsynaptic receptor clusters to the release of a single synaptic vesicle. Here, we observed that the α1-antibody treated striatal neurons exhibited a lower mIPSC frequency, 0.46 ± 0.14 Hz, compared with cells treated with a control-antibody, 1.42 ± 0.32 Hz, however the frequency was not significantly different compared with untreated cells, 0.89 ± 0.20 Hz, although it did show a trend toward reduction (*p* = 0.0149, Kruskal–Wallis; Fig. 3*K*,*L*). In addition, a trend toward a decrease in mIPSC amplitude was observed (untreated 34 ± 3 pA, control-antibody 36 ± 3 pA, α1-antibody 25 ± 3 pA; Fig. 3*M*), but no differences in rise time, half width, or decay time (data not shown) were observed. A reduction in both amplitude and frequency indicates a loss of functional receptors from the synapse, as well as perhaps the loss of functional inhibitory synapses after the 24-h antibody treatment. These conclusions are consistent with the initial observations from our immunocytochemical experiments. Intriguingly, for autaptic striatal neurons treated with 1 μg of the α1γ2-antibody for 24 h, we did not detect any changes in the total or synaptic GABA currents (Fig. 3*B*,*C*,*E*,*F*), suggesting that this antibody has little or no direct effect on GABA_A_Rs on these cells.

### Cortical-striatal neuron network activity increases after a 24-h treatment with α1-antibody but not the α1γ2-antibody

Another fundamental question particularly relevant to the etiology of this form of encephalitis is whether such autoantibodies alter the properties of cortical striatal networks. Specifically, we sought to test the hypothesis that by reducing GABA currents, these antibodies would lead to hyper-excitable network activity, explaining in part why many GABA_A_RE patients display seizures.

**Figure 4.**
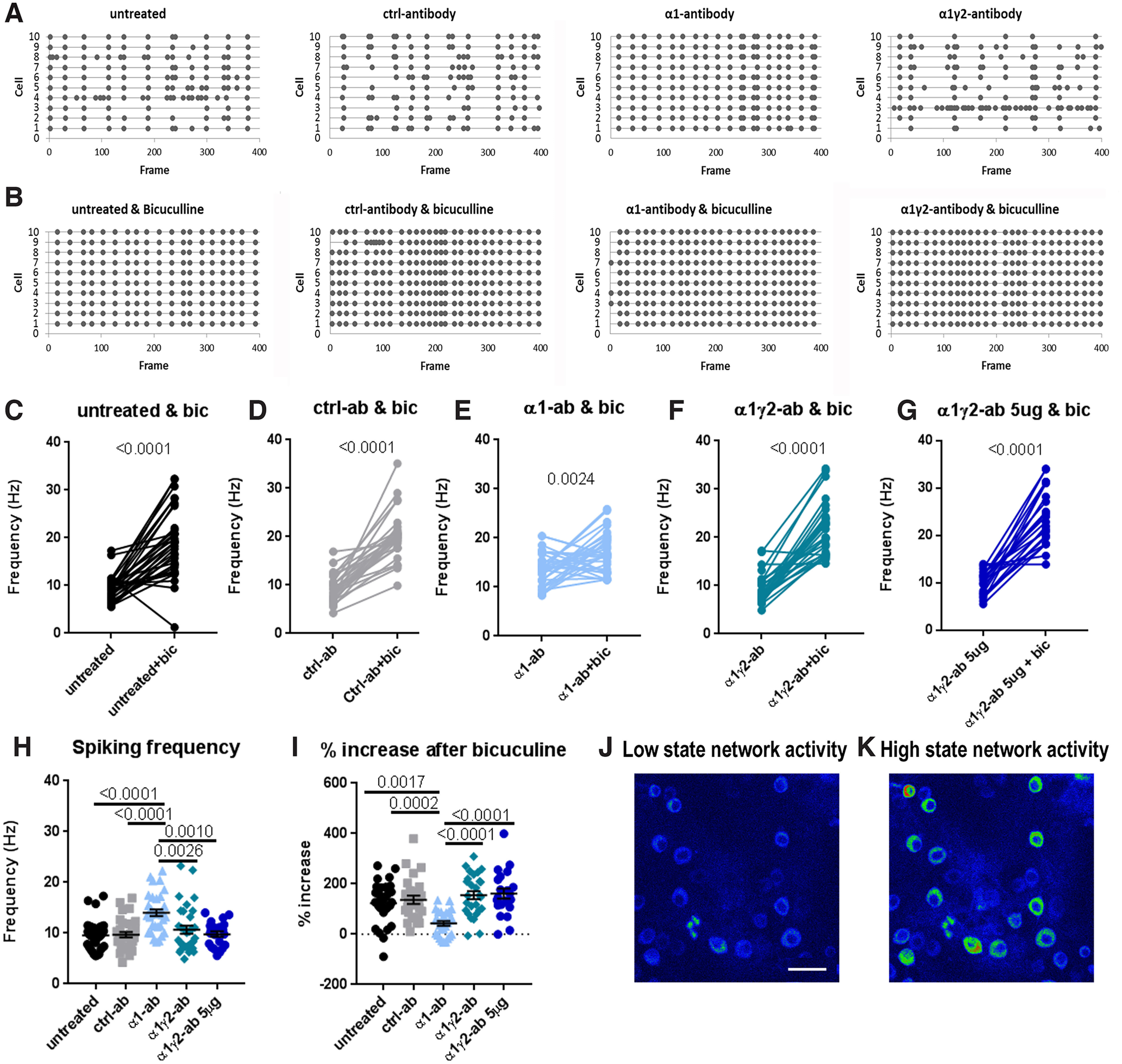
Cortical-striatal neuron network activity increases after a 24 h treatment with α1-antibody but not the α1γ2-antibody. Examples of network activity, based on the frequency of calcium transients, 24 h after antibody treatment (***A***) and directly after the addition of 30 μm bicuculline (***B***). ***C–G***, The average spiking frequency per field of view per condition shows that the addition of bicuculline leads to higher average spike frequency for all conditions, untreated 9.30 ± 0.47 Hz versus bicuculline 18.86 ± 1.22 Hz, *t*_(32)_ = 7.64, *p* < 0.0001; control-antibody 9.17 ± 0.55 Hz versus bicuculline 20.18 ± 0.99 Hz, *t*_(26)_ = 10.34, *p* < 0.0001; α1-antibody 13.76 ± 0.58 Hz versus bicuculline 17.05 ± 0.69, Hz *t*_(32)_ = 3.29, *p* = 0.0024; α1γ2-antibody 9.30 ± 7.61 Hz versus bicuculline 21.91 ± 1.08 Hz, *t*_(26)_ = 11.04, *p* < 0.0001; α1γ2-antibody 5 μg 9.79 ± 0.54 Hz versus bicuculline 23.9 ± 1.31 Hz, *t*_(20)_ = 9.80, *p* < 0.0001; all paired *t* tests. ***H***, However, under conditions treated with α1-antibody for 24 h (***A***) spiking starts off with a higher average frequency, *H*_(4)_ = 29.62, *p* < 0.0001, Kruskal–Wallis; untreated 9.58 ± 0.47 Hz versus α1-antibody 14.03 ± 0.66 Hz *p* < 0.0001, control 9.68 ± 0.53 Hz versus α1-antibody *p* < 0.0001, α1-antibody versus α1γ2-antibody 10.68 ± 0.79 Hz *p* = 0.0010, α1-antibody versus α1γ2-antibody 5 μg 9.79 ± 0.54 *p* = 0.0026, (***I***) and shows a smaller percentage increase after the addition of bicuculline compared with other conditions, *F*_(4,127)_ = 9.46, *p* < 0.0001, ANOVA; untreated 123.5 ± 15.01% versus α1-antibody 42.7 ± 9.66% *p* = 0.0017, control 136.6 ± 16.16% versus α1-antibody *p* = 0.0002, α1-antibody versus α1γ2-antibody 155 ± 16.01% *p* < 0.0001, α1-antibody versus α1γ2-antibody 5 μg 160.8 ± 20.02% *p* < 0.0001. Example images of a low (***J***) and high (***K***) network spiking activity; scale bar: 40 μm. Each data point represents the average spiking activity of one ROI. Error bars represent SEM.

To test this concept, we designed several calcium imaging experiments in which cultured cortical/striatal neurons were initially infected at four DIV with a lentivirus expressing jRCamP1b (under the Synapsin promoter; Dana et al., 2016), a genetically encoded calcium indicator, and allowed to grow for 10 more days (14 DIV) before antibody treatment. Cultures were then treated for 24 h with either α1-antibody, α1γ2-antibody, or control-antibody, or left untreated before being subjected to live imaging to monitor neuronal spiking activity by detecting somatic calcium transients of individual cells. Subsequently, we added bicuculline (30 μm; a GABA_A_R antagonist) and re-imaged the activity of the same neurons for a within-cell comparison (Fig. 4*A*,*B*). This was used to reveal whether additional inhibitory drive is present in the system after antibody treatment.

When we looked at the excitability of the network, we found that the presence of the α1-antibody for 24 h led to a significantly higher spiking frequency compared with both control-antibody and untreated cultures (*p* < 0.0001, Kruskal–Wallis; untreated 9.58 ± 0.47 Hz, α1-antibody 14.03 ± 0.66 Hz, control-antibody 9.68 ± 0.53 Hz; Fig. 4*A*,*H*). This is consistent with its negative impact on GABA-mediated currents seen in autaptic striatal neurons (Fig. 3). This finding raised the question how much of the inhibitory drive was still present in the network after 24-h α1-antibody treatment. To address this question, we added 30 μm bicuculline to the network. The addition of bicuculline led to an increase in network spiking activity in the untreated and control-antibody conditions (Fig. 4*C–G*), but had minimal effects on cultures treated with α1-antibody for 24 h (*p* < 0.0001, ANOVA; untreated 123.5 ± 15.01% compared with control, α1-antibody 42.7 ± 9.66%, and control-antibody 136.6 ± 16.16%; Fig. 4*E*,*I*). These data indicate that most available GABA_A_R receptors are already affected by the α1-antibody, leading to a network with a small remaining inhibitory drive.

In cultures treated with the α1γ2-antibody, we did not observe any effect on neuronal spiking activity in these networks compared with either untreated or control-antibody-treated cultures (Fig. 4*H*,*F*,*I*). Since the α1γ2-antibody appears to have a different antibody avidity compared with the α1-antibody (Kreye et al., 2021), we also investigated whether a higher concentration of this antibody (5 μg/ml instead of 1 μg/ml) would have an effect. Here again, no change in network spiking was detected (Fig. 4*G–I*), further supporting the concept that this antibody has no overt direct effects on GABA_A_Rs or neuronal spiking in these cultured neurons.

### α1-antibody elicits rapid effects on GABA_A_ receptor function

**Figure 5.**
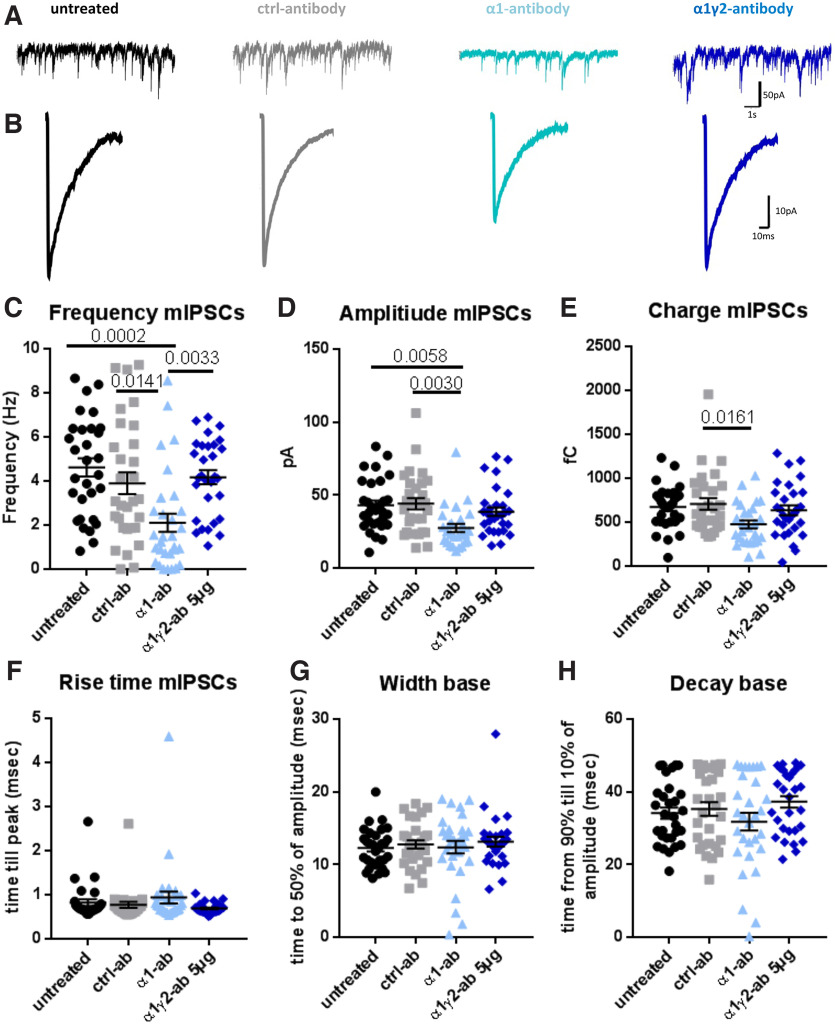
α1-antibody but not α1γ2-antibody reduce GABA currents after a 1-h incubation. Example traces of mIPSC frequency (***A***) and amplitude (***B***). ***C***, mIPSCs recorded 1 h after antibody treatment show reduction in frequency, *F*_(3,116)_ = 7.10, *p* = 0.0002, ANOVA; untreated 4.62 ± 0.41 Hz versus α1-antibody 2.10 ± 0.41 Hz *p* = 0.0002, control 3.90 ± 0.50 Hz versus α1-antibody *p* = 0.0141, α1-antibody versus α1γ2-antibody 4.18 ± 0.32 Hz *p* = 0.0033, (***D***) and amplitude for the α1-antibody compared with other groups, *F*_(3,112)_ = 5.27 *p* = 0.0020, ANOVA; untreated 42.97 ± 3.24 pA versus α1-antibody 27.45 ± 2.71 pA *p* = 0.0058, control 44.03 ± 3.87 pA versus α1-antibody *p* = 0.0030. ***E***, mIPSC charge only showed a difference between α1 and control-antibody, *F*_(3,112)_ = 3.52, *p* = 0.0174, ANOVA; control 707.3 ± 64.83 fC versus α1-antibody 475.5 ± 45.19 fC *p* = 0.0161. ***F***, However, rise time, *H*_(3)_ = 4.22, *p* = 0.2385, Kruskal–Wallis, (***G***) half width, *H*_(3)_ = 1.23, *p* = 0.7452, Kruskal–Wallis, (***H***) and decay time, even for the α1-antibody, were not affected, *H*_(3)_ = 2.79, *p* = 0.425, Kruskal–Wallis. Data points represent one cell each. Error bars represent SEM.

The dramatic effect of the α1-antibody on cortical-striatal networks raises fundamental questions of how this antibody changes the functionality of these receptors. Possibilities include direct actions on these receptors and/or their internalization. For some neurotransmitter receptors, antibodies have been found to stimulate their internalization on a time scale of a few hours. For example, antibodies targeting NMDAR, present in the CSF of patients with NMDAR encephalitis, showed reductions in receptor surface levels in the first 2–4 h with internalization peaking after 12 h (Moscato et al., 2014). We thus examined whether changes in GABA-mediated currents by the α1-antibody seen at 24 h could also occur on shorter time scales. This was initially accomplished by performing whole-cell patch recording of mIPSCs in cortico-striatal mass-cultures 1 h after the addition of 1 μg/ml of the α1-antibody. Interestingly, at this time, we observed a dramatic decrease in the frequency (*p* = 0.0002, ANOVA; untreated 4.62 ± 0.41 Hz, α1-antibody 2.10 ± 0.41 Hz, and control-antibody 3.90 ± 0.50 Hz; Fig. 5*A*,*C*), charge (*p* = 0.0174, ANOVA; control 707.3 ± 64.83 fC, α1-antibody 475.5 ± 45.19 fC; Fig. 5*E*) and amplitude (*p* = 0.0020, ANOVA; untreated 42.97 ± 3.24 pA, α1-antibody 27.45 ± 2.71 pA, and control-antibody 44.03 ± 3.87 pA; Fig. 5*B*,*D*) of mIPSCs in cultures treated with the α1-antibody as compared with untreated or control-antibody treated cultures. Of note, there was no observed effect of the α1-antibody on mIPSC kinetics including their rise time, half-width, or decay times (Fig. 5*F–H*). Reduction in both frequency and amplitude could indicate a reduction in the number of receptors at synapses, which would cause the mIPSCs to fall below detection levels. Interestingly, the observed effects on mIPSCs were not pronounced after a 24-h antibody incubation (Fig. 3*K–M*), reflecting possible homeostatic exchange in synaptic GABA_A_R subunit composition in these neurons. Of note the addition of the α1γ2-antibody at 5 μg/ml for 1 h had no detectable effect on mIPSCs frequency and amplitude or kinetics (Fig. 5*A–H*).

**Figure 6.**
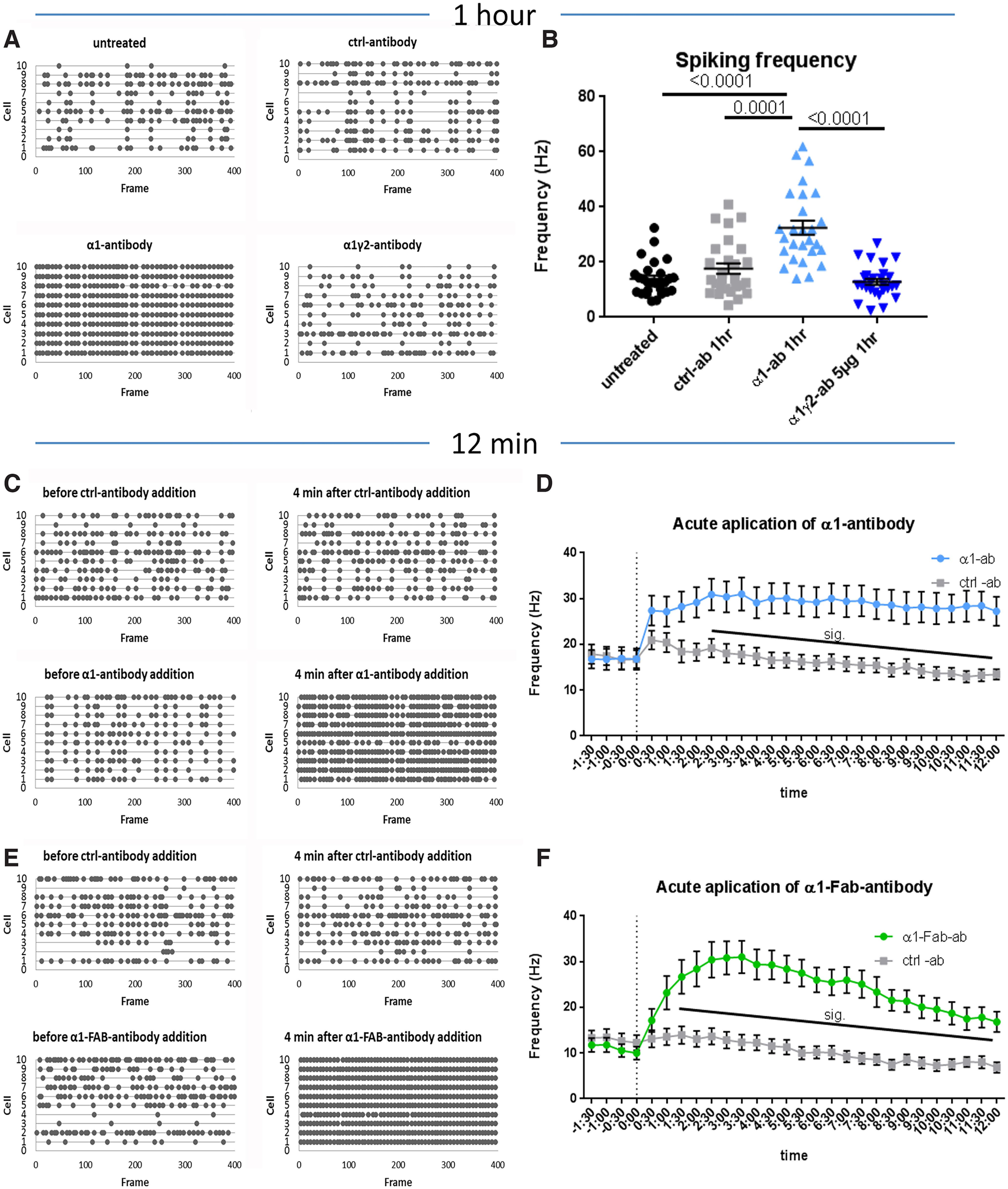
α1-antibody elicits rapid effects on cortical-striatal calcium transients. ***A***, Example spike frequency plots and quantification (***B***) of network spiking activity shows that 1 h after α1-antibody is added, activity is increased compared with other conditions, *H*_(3)_ = 40.12, *p* < 0.0001, Kruskal–Wallis; untreated 13.98 ± 1.21 Hz versus α1-antibody 32.46 ± 2.58 Hz *p* < 0.0001, control 17.64 ± 1.93 Hz versus α1-antibody *p* = 0.0001, α1-antibody versus α1γ2-antibody 5 μg 12.87 ± 1.13 Hz *p* < 0.0001. This effect was not reversible by antibody washout (Extended Data Fig. 6-1). When the α1-antibody is added for only 12 min to cortical-striatal cultures (***C***, example plots), there is a significant increase in spiking frequency, already after 2 min, compared with other conditions (***D***) that is sustained for the full 12 min, *F*_(1,29)_ = 10.82, *p* = 0.0026, repeated measures two-way ANOVA. The dotted vertical line indicates when antibody is added to the culture. Addition of an Fab fragment from the α1-antibody (***E***) similarly induces an increase in spike frequency that keeps rising up till 4 min after addition, even higher than the full-length antibody, and then slowly declines, although not all the way back to control level over the course of 12 min (***F***), *F*_(1,33)_ = 18.27, *p* = 0.0002, repeated measures two-way ANOVA. Each data point represents the average spiking activity of one field of view. Error bars represent SEM.

To complement these recordings, we also added these antibodies to mass-cultures and monitored firing frequency by calcium imaging. Here, we found that network excitability was dramatically increased in the presence of the α1-antibody after only 1 h, but not in untreated cultures or those treated with control-antibody (*p* < 0.0001, Kruskal–Wallis; untreated 13.98 ± 1.21 Hz, α1-antibody 32.46 ± 2.58 Hz, control-antibody 17.64 ± 1.93 Hz, and α1γ2-antibody 5 μg 12.87 ± 1.13 Hz *p* < 0.0001; Fig. 6*A*,*B*). This increase in spiking activity was not reversible by washing out of the α1-antibody (Extended Data Fig. 6-1). Importantly, the addition of the α1γ2-antibody at higher concentrations (5 μg/ml) for 1 h had no effect on the network spiking activity (Fig. 6*B*).

10.1523/ENEURO.0369-22.2022.f6-1 Extended Data Figure 6-1 Effects of α1-antibody 6 h after antibody washout. Example of neurons stained for MAP2 (white), α1-antibody (red), VGAT (blue), and Merge (α1-antibody and VGAT) 6 h after antibody washout (***A***). Example spike plots of untreated (***B***) and α1-antibody-treated (***C***) cortical-striatal cultures during calcium imaging experiments. ***D***, Spiking frequency is still increased 6 h after α1-antibody washout *t*_(52)_ = 11.51, *p* < 0.0001, *t* test, untreated 12.24 ± 0.98 Hz, α1-antibody 36.71 ± 1.88 Hz. ***E***, Example mIPSC traces. Even 6 h after α1-antibody removal, we see decreases in frequency *t*_(58)_ = 3.838, *p* = 0.0003, *t* test, untreated 3.35 ± 0.36 Hz, α1-antibody 1.56 ± 0.30 Hz, (***F***) amplitude *t*_(46.71)_ = 4.44, *p* < 0.0001, Welch’s *t* test, untreated 36.65 ± 2.86 pA, α1-antibody 21.93 ± 1.68 pA, (***G***) charge *t*_(57)_ = 4.77, *p* < 0.0001, *t* test, untreated 655.8 ± 53.81 fC, α1-antibody 345.6 ± 35.69 fC, (***H***) half-width *t*_(58)_ = 2.426, *p* = 0.0184, *t* test, untreated 13.35 ± 0.46 ms, α1-antibody 11.26 ± 0.73 ms, (***J***) and decay time *t*_(58)_ = 3.414, *p* = 0.0012, *t* test, untreated 37.57 ± 1.38 ms, α1-antibody 29.07 ± 2.07 ms (***K***), and an increase in rise time *t*_(30.38)_ = 2.976, *p* = 0.0057, Welch’s *t* test, untreated 0.72 ± 0.03 ms, α1-antibody 1.2 ± 0.16 ms (***I***). Download Figure 6-1, TIF file.

Conceptually, the faster effects observed for the α1-antibody on GABA currents and network activity could be caused by two possible mechanisms. The first is that the α1-antibody has a direct effect on the receptor, either via an allosteric interaction or as a competitive antagonist to GABA. The second is that the antibody promotes receptor crosslinking and triggers internalization. To distinguish between these possible mechanisms, we performed two types of experiments.

In the first, we monitored neuronal spiking activity by calcium imaging before and then continuously for 12 min after the addition of the antibody. Here, we observed a rapid (<5 min) increase in spiking frequency of cells treated with α1-antibody that was sustained during the remaining 12 min of the imaging session (*p* = 0.0026, repeated measures two-way ANOVA; Fig. 6*C*,*D*). This increase was not seen for the control-antibody (Fig. 6*C*,*D*), suggesting that the antibody has an effect on neuronal firing already after minutes.

To rule out fast internalization through α1-antibody-mediated receptor crosslinking, we employed a Fab-fragment from the α1-antibody (Kreye et al., 2021). When 2 μg/ml of the Fab-fragment was added to our cortical/striatal cultures, we observed a rapid increase in network activity similar to that seen with the α1-antibody, reaching a peak at 4 min after antibody addition (*p* = 0.0002, repeated measures two-way ANOVA; Fig. 6*E*,*F*), which remained elevated for the duration of the imaging session compared with the control-antibody (Fig. 6*F*). These results strongly hint at a direct effect of the α1-antibody, allosteric or antagonistic, on GABA_A_R containing this subunit rather than loss of function by rapid receptor internalization.

To explore whether the effects of the α1-antibody are reversible, we performed both calcium imaging and electrophysiological experiments on cortical-striatal cultures incubated for only 1 h with the α1-antibody before being washed out. When neurons were stained for the antibody and VGAT, 6 h after washout, we saw that the antibody was still present on the neurons at synapses (Extended Data Fig. 6-1*A*). Monitoring spike frequency of untreated and α1-antibody treated cortical-striatal cultures revealed a dramatic increase in firing frequency 6 h after washout (Extended Data Fig. 6-1*B*,*D*; *p* < 0.0001, *t* test, untreated 12.24 ± 0.98 Hz, α1-antibody 36.71 ± 1.88 Hz), indicating the antibody remains bound and continues to repress GABA_A_ receptor-mediated inhibition in these networks. To assess whether part of this effect is because of an ongoing block of synaptic GABA_A_ receptor, we recorded the frequency, amplitude, charge and kinetics of the mIPSC 6 h after washout. Here, we also observed a remarkable decrease in mIPSC frequency (*p* = 0.0003, *t* test, untreated 3.35 ± 0.36 Hz, α1-antibody 1.56 ± 0.30 Hz), amplitude (*p* < 0.0001, Welch’s *t* test, untreated 36.65 ± 2.86 pA, α1-antibody 21.93 ± 1.68 pA) and charge (*p* < 0.0001, *t* test, untreated 655.8 ± 53.81 fC, α1-antibody 345.6 ± 35.69 fC; Extended Data Fig. 6-1*E–H*). We also detected a decrease in the decay time (*p* = 0.0012, *t* test, untreated 37.57 ± 1.38 ms, α1-antibody 29.07 ± 2.07 ms), half-width of these minis (*p* = 0.0184, *t* test, untreated 13.35 ± 0.46 ms, α1-antibody 11.26 ± 0.73 ms), as well as an increase in-rise time (*p* = 0.0057, Welch’s *t* test, untreated 0.72 ± 0.03 ms, α1-antibody 1.2 ± 0.16 ms; Extended Data Fig. 6-1*I–K*). Together, these data indicate that the α1-antibody after washout remains bound to receptors and continues to repress synaptic GABA_A_ receptor-mediated responses as well as the inhibitor drive in these networks. The change in kinetics of these minis suggests either direct effects of the α1-antibody on α1-containing receptors or the emergence/unmasking of non-α1-containing receptors with different kinetic properties at these synapses.

### α1γ2-antibody does not affect GABA_A_ receptor function by interference with their benzodiazepine site

**Figure 7.**
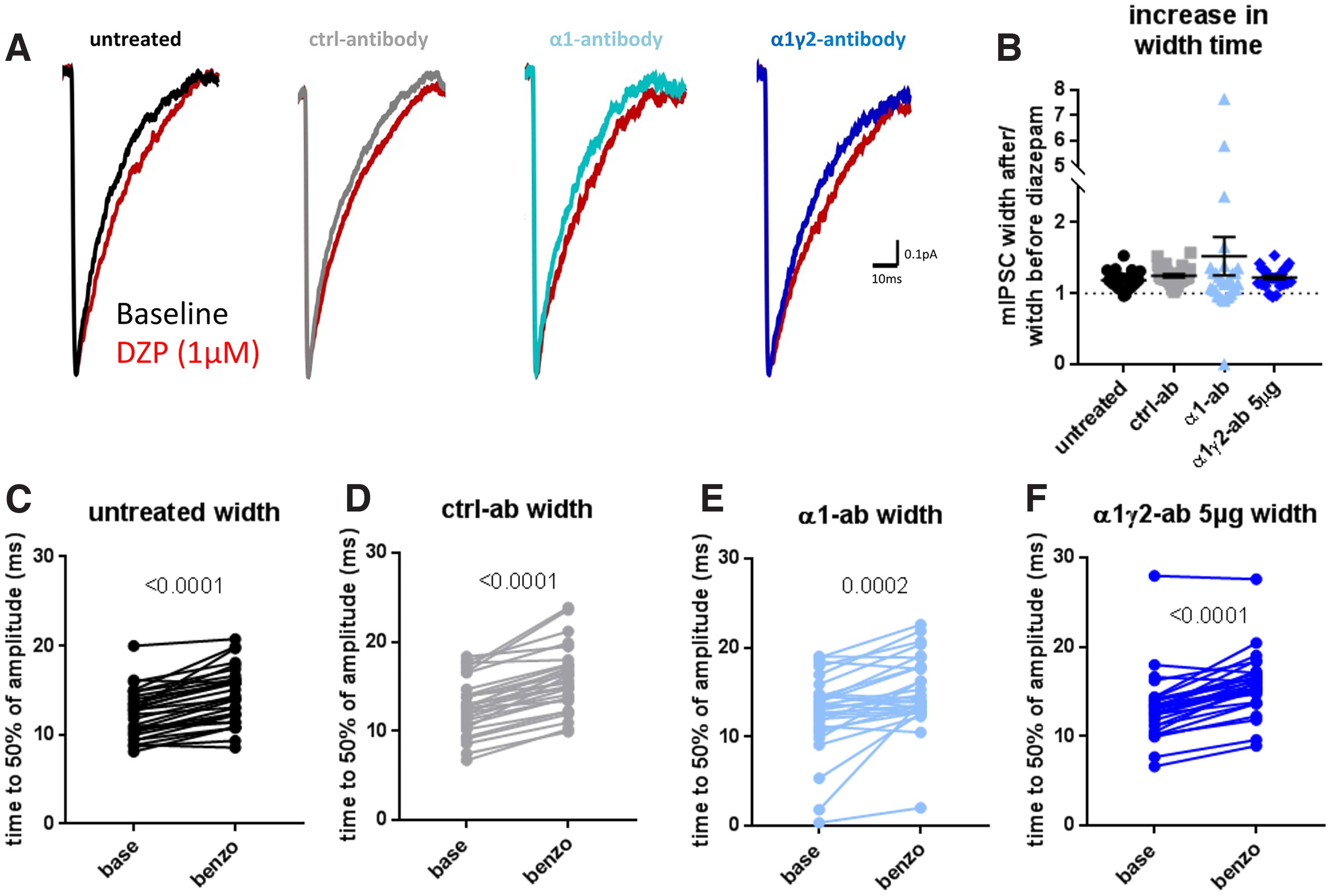
α1-antibody and α1γ2-antibody do not alter the effect of benzodiazepine. Example traces (***A***) and quantification (***B–F***) show that in all conditions, the addition of 1 μm diazepam (DZP) is still able to increase the half width of mIPSCs. ***C–F***, show the individual increase in half width of each cell per condition [untreated (***C***): *t*_(29)_ = 7.991, *p* < 0.0001, paired *t* test; untreated base 12.27 ± 0.49 ms, untreated benzo 14.46 ± 0.57 ms; control-antibody (***D***): *t*_(28)_ = 10.32, *p* < 0.0001, paired *t* test; control-antibody base 12.77 ± 0.57 ms, control-antibody benzo 15.74 ± 0.66 ms; α1-antibody (***E***): *t*_(28)_ = 4.30, *p* = 0.0002, paired *t* test; α1-antibody base 12.69 ± 0.82 ms, α1-antibody benzo 15.08 ± 0.75 ms; α1γ2-antibody (***F***): *t*_(29)_ = 8.73, *p* < 0.0001, paired *t* test; α1γ2-antibody base 13.14 ± 0.67 ms, α1γ2-antibody benzo 15.8 ± 0.61 ms]. ***B***, Quantitation of half-widths ratio (calculated as halfwidth with diazepine/halfwidth baseline). A value above 1 indicates an increase (*H*_(3)_ = 5.03, *p* = 0.1694, Kruskal–Wallis; untreated 1.18 ± 0.02, control-antibody 1.25 ± 0.02, α1-antibody 1.52 ± 0.27, α1γ2-antibody 1.22 ± 0.02). Each data point represents the average half-width of mIPSCs per cell. Error bars represent SEM.

In the experiments presented thus far, we failed to detect any remarkable effects of the α1γ2-antibody on GABA currents and/or distribution of these receptors in striatal neurons. This lack of response is even more interesting in the light of a previous study wherein the α1γ2-antibody exhibited a strong epileptogenic effect in rodents when the antibody was intraventricularly delivered via an osmotic Alzet pump (Kreye et al., 2021). This raised the question as to how this antibody can affect neuronal excitability *in vivo* but not in the used *in vitro* systems. As the α1γ2-antibody binds an epitope at the α1 and γ2 subunit interface, it is conceptually possible that this antibody interferes with the binding of endogenous benzodiazepine like compounds (e.g., endozepines), known to act at a site situated between the α and γ subunit of GABA receptor subtypes containing α1, α2, α3, and α5 subunits. To explore this possibility, we recorded mIPSCs in mass-cortical-striatal co-cultures, 1 h after the addition of the α1γ2-antibody in the presence and absence of the benzodiazepine, diazepam. We chose to add the diazepam after 1 h of antibody incubation because this way we could measure whether the α1γ2-antibody would silently bind the GABA_A_R and thereby prevent the diazepam from binding, rather than a 24-h time point at which there could be confounding effects caused by subunit substitution or similar homeostatic effects. When mIPSCs were recorded in untreated neurons, the half-width times and the charge were observed to increase, as expected, in the presence of the diazepam (*p* < 0.0001, paired *t* test; untreated base 12.27 ± 0.49 ms, untreated benzo 14.46 ± 0.57 ms; Fig. 7*A*,*C*). In cultures treated with either the α1γ2-antibody, α1-antibody, or control-antibody, diazepam was also able to increase the half-width times of mIPSC [*p* < 0.0001, paired *t* test; control-antibody base 12.77 ± 0.57 ms, control-antibody benzo 15.74 ± 0.66 ms (Fig. 7*D*); *p* = 0.0002, paired *t* test; α1-antibody base 12.69 ± 0.82 ms, α1-antibody benzo 15.08 ± 0.75 ms (Fig. 7*E*); *p* < 0.0001, paired *t* test; α1γ2-antibody base 13.14 ± 0.67 ms, α1γ2-antibody benzo 15.8 ± 0.61 ms (Fig. 7*F*)]. When we compared the relative increase in mIPSCs charge or half-width (ratio of after/before addition of diazepam), we see that all conditions respond in a similar manner to the addition of diazepam (Fig. 7*B*). These results indicate that the actions of diazepam are not impaired by either the α1γ2-antibody or α1-antibody and that neither directly interferes with the benzodiazepine binding site.

**Figure 8.**
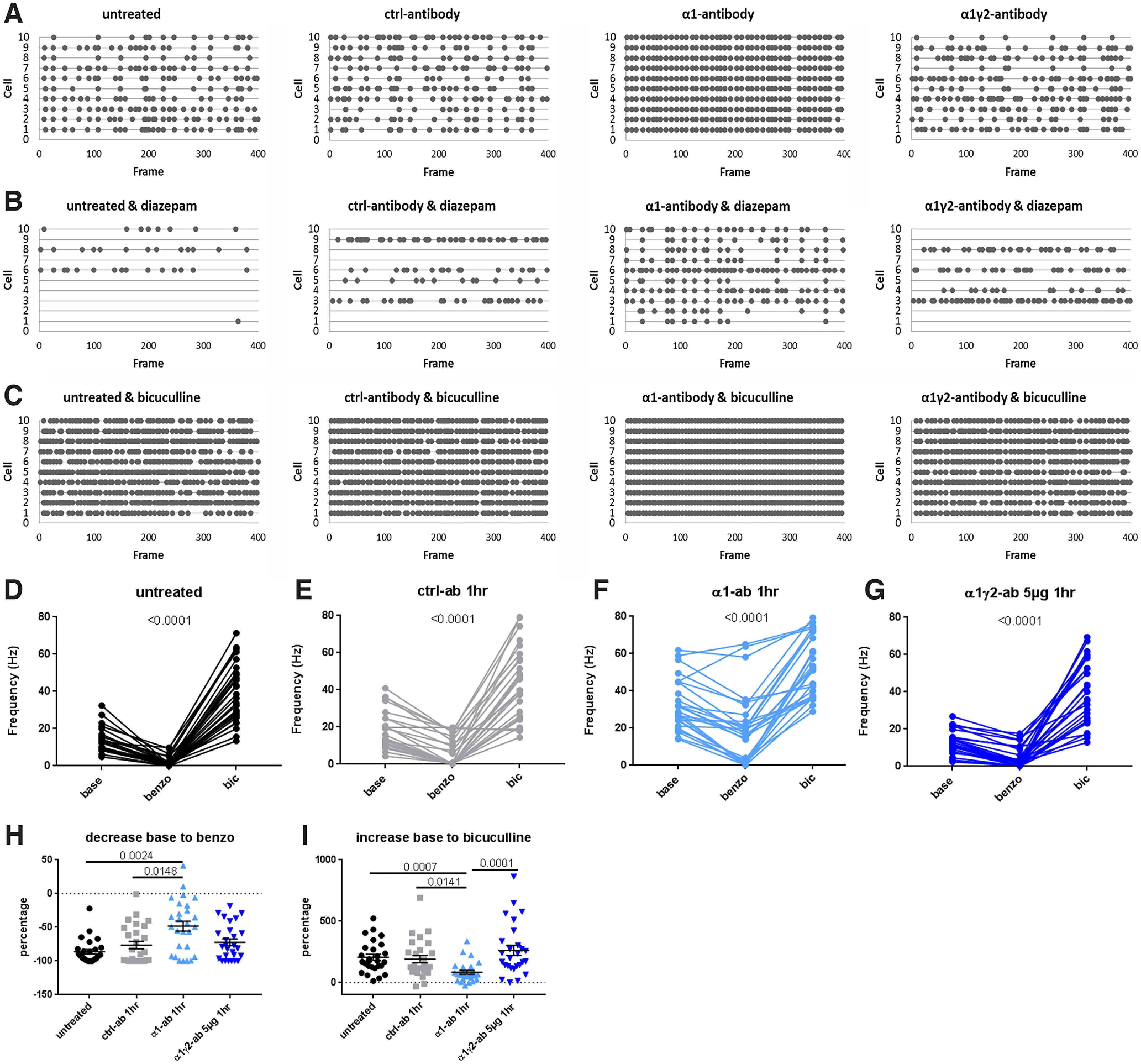
Difference in network reaction to diazepam when treated with the α1-antibody but not α1γ2-antibody. Example traces of calcium transient spiking activity 1 h after the addition of α1-antibody, α1γ2-antibody, or control-antibody (***A***). Example traces of network spiking activity after addition of 1 μm diazepam to the cultures shown in ***A*** (***B***). Example traces of network spiking activity after addition of 30 μm bicuculline to the cultures shown in ***B*** (***C***). ***D–G***, Average spiking activity in each field of view (∼15 cells) in baseline, diazepam, and bicuculline condition for each experimental group shows spiking responses to these drugs [untreated (***D***): *F*_(2,52)_ = 129.8, *p* < 0.0001, repeated measures ANOVA; base 13.94 ± 1.22 Hz vs benzo 2.03 ± 0.53 Hz *p* < 0.0001, base vs bic 38.93 ± 3.04 Hz *p* < 0.0001, benzo vs bic <0.0001; control-antibody (***E***): *F*_(2,52)_ = 91.27, *p* < 0.0001 repeated measures ANOVA; base 17.64 ± 1.93 Hz vs benzo 5.78 ± 1.44 Hz *p* = 0.0003, base vs bic 43.08 ± 3.70 Hz *p* < 0.0001, benzo vs bic *p* < 0.0001; α1-antibody (***F***): *F*_(2,52)_ = 67.03, *p* < 0.0001, repeated measures ANOVA; base 32.46 ± 2.58 Hz vs benzo 19.7 ± 3.64 Hz *p* = 0.0001, base vs bic 52.87 ± 3.14 Hz *p* < 0.0001, benzo vs bic *p* < 0.0001; α1γ2-antibody 5 μg (***G***): *F*_(2,52)_ = 94.32, *p* < 0.0001, repeated measures ANOVA; base 12.87 ± 1.16 Hz vs benzo 4.64 ± 1.06 Hz *p* = 0.0084, base vs bic 39.38 ± 3.34 Hz *p* < 0.0001, benzo vs bic *p* < 0.0001]. ***H***, Quantification of percent changes in spiking activity following the addition of 1 μm diazepam. Here, diazepam leads to a ∼75% reduction in spiking activity compared with cultures without diazepam (Fig. 6*B*), with the exception to the α1-antibody condition where the reduction was significantly smaller (∼50%) *H*_(3)_ = 14.67, *p* = 0.0021, Kruskal–Wallis; untreated −86.29 ± 3.37% versus α1-antibody −48.32 ± 7.49% *p* = 0.0024, control −76.37 ± 5.46% versus α1-antibody *p* = 0.0148. ***I***, Quantification of percent changes in spiking activity following the addition of 30 μm bicuculline. This leads to an ∼200% increase in network activity compared with baseline (Fig. 6*B*) for all groups except for α1-antibody condition, where the increase is only 80%, *H*_(3)_ = 22.77, *p* < 0.0001, Kruskal–Wallis; untreated versus α1-antibody *p* = 0.0007, control versus α1-antibody *p* = 0.0141, α1-antibody versus α1γ2-antibody *p* < 0.0001. Error bars represent SEM.

To further investigate possible effects of these antibodies on the actions of benzodiazepine at a network level, we performed calcium imaging experiments, 1 h after antibody incubation. With the addition of 1 μm diazepam, the spiking activity of α1γ2 treated cultures went down by −72.12 ± 5.05% (Fig. 8*B*,*H*) compared with cultures to which only control-antibody was added (Fig. 6*B*). This effect was also seen in cultures treated with the α1-antibody. However, here the average firing frequency remained higher, with only a −48.32 ± 7.49% decrease compared with baseline (*p* = 0.0021, Kruskal–Wallis; untreated −86.29 ± 3.37%, control-antibody −76.37 ± 5.46%; Fig. 8*B*,*H*). As expected, adding 30 μm bicuculline triggered an increase in spiking activity for all four conditions compared with spiking activity during diazepam incubation (8D-G). Interestingly, cultures treated with the α1γ2-antibody returned to spiking levels (262.2 ± 40.68%) similar to untreated (205.5 ± 24.71%) and control-antibody (190.7 ± 29.85%), whereas the α1-antibody showed a smaller increase in activity (82.54 ± 15.76%) compared with untreated, α1γ2-antibody and control-antibody conditions (*p* < 0.0001, Kruskal–Wallis; Fig. 8*I*). These results indicate that the increases in neuronal excitability *in vivo* (Kreye et al., 2021) associated with the addition of α1γ2-antibody is not necessarily due its ability to block the benzodiazepine-binding-site of GABA_A_ receptors containing either α1 or α1γ2 subunits. However, this conclusion requires some caution as the continued effect of diazepam on these antibody-treated cultures could be because of other benzo-sensitive GABA_A_ receptors containing, e.g., γ2 and α2, α3, or α5 subunits. Clearly, single channel recording would be necessary to resolve this issue.

## Discussion

Autoimmune encephalitis is a devastating disorder associated with antibodies targeting neural-specific antigens. While these antibodies target one primary antigen, evidence exists that polyclonal antibodies react to multiple targets/receptors with different subunit compositions in a single patient (Kreye et al., 2021). This complexity makes it difficult to define causality between a specific antibody and a patient’s symptoms, and/or whether they operate via similar or distinct modes of action. To bring some clarity to these issues, we have characterized the molecular and functional modes of action of two recently isolated monoclonal antibodies (Kreye et al., 2021) from a GABA_A_RE patient (Nikolaus et al., 2018), one targeting the α1 subunit and another one targeting the α1γ2 subunits of GABA_A_Rs. The former was found to have long-term effects on receptor distribution and fast inhibitory effects on GABA_A_R function that did not require internalization by antibody cross-linking. Intriguingly, we could detect no overt functional effects on GABA_A_Rs by the α1γ2-antibody, although *in vivo*, it was previously found to elicit seizure activity (Kreye et al., 2021), implying it may have indirect effects on receptor function via, e.g., microglia. These observations highlight diverse modes of action of autoantibodies, contributing mechanistically to autoimmune encephalitis.

### Multimodal effects of the α1-antibody

Our analysis of the α1-antibody indicates that it acts on several levels of GABA_A_R function, including both long-term and short-term effects. Long-term addition of the α1-antibody (∼24 h) to primary cortical/striatal co-cultures promotes a loss of immune-positive receptors from GABAergic VGAT-labeled synapses (Fig. 1). We found that under basal conditions ∼80% of GABAergic synapses between cortical/striatal neurons possess α1-containing GABA_A_Rs. This was reduced to 50% following 24-h antibody incubation. Autoantibody-mediated redistribution of receptors has also been reported for other antigen/receptors (Prüss, 2021) and is consistent with antibody induced movement of receptors away from the synapse and/or their internalization (Ohkawa et al., 2014; Petit-Pedrol et al., 2014; Pettingill et al., 2015).

Using whole-cell patch recording of striatal autaptic neurons, we also explored the functional impact of the α1-antibody on both synaptic and extrasynaptic GABA_A_R-mediated inhibitory currents following a 24-h treatment. Here, we observed a dramatic decrease in total inhibitory GABA currents, with no effect on excitatory currents (Fig. 3). This was associated with a remarkable decrease in evoked IPSCs as well as an increase in decay kinetics. These latter data are consistent with loss of synaptic α1-GABA_A_Rs and a possible change in synaptic subunit composition. Sucrose and mIPSC recordings support these conclusions, as they also reveal a decrease in total inhibitory synaptic currents (sucrose response) and number of functional synapses (frequency) with no change in mIPSC amplitude of the remaining synaptic receptors (Fig. 3).

To distinguish between the real-time effects of the α1-antibody and homeostatic changes such as receptor subunit composition, we assessed changes in synaptic GABA_A_R function following a shorter 1-h incubation with cortical/striatal co-cultures. Here, we observed a dramatic decrease in mIPSC frequency, amplitude, and charge (Fig. 5*C–E*) with no change in rise time, half-width, or decay times (Fig. 5*F–H*), indicating a possible direct inhibition of GABA_A_Rs. These latter data further support the concept that long-term exposure of neurons to the α1-antibody triggers homeostatic changes to neurons, including possible changes in receptor subunit composition to α2-containing, α3-containing, or α5-containing receptors. Of note these other GABAAR subunits have slower decay kinetics than the α1 subunit (Gingrich et al., 1995). As such redistribution of α1-containing receptors away from the synapse at later time points could account for the observed increase in decay times after the 24-h antibody incubation, which have yet to occur after 1 h of incubation. Interestingly, changes in receptor subunit composition are also observed in networks after long-term exposure to allosteric agonists such as benzodiazepine (Vinkers et al., 2012).

Further hints to the mode of impact of the α1-antibody came from examining its effects on network spiking activity of cortical-striatal co-cultures using calcium imaging. Consistent with its ability to decrease GABA mediate currents, we observed dramatic increases in spiking frequency following the addition of the α1-antibody for 1 or 24 h (Figs. 4, 6*A*,*B*). Another interesting observation is that after the 1-h treatment, these networks were less sensitive to benzodiazepine regulation. This is also observed in GABA_A_RE patients, who generally present with status epilepticus (O’Connor et al., 2019) that does not respond to treatment with benzodiazepines (Rossetti and Lowenstein, 2011). Remarkably, when the α1-antibody was added acutely to the network, we could detect real-time changes in neuronal spiking activity in as little as 2–3 min (Fig. 6*C*,*D*), indicating that this antibody can directly modify α1-containing GABA_A_Rs function in a fast manner. This appears to be independent of a fast antibody-mediated cross-linking and receptor internalization as the acute application of a noncross-linking Fab-fragment of the α1-antibody also triggered a real-time increase in spiking activity in ∼2 min (Fig. 6*E*,*F*) that is not quickly washed out (Extended Data Fig. 6-1). Future, biophysical studies and pHluorin-based imaging of tagged receptors will be necessary to resolve these options.

Taken together, these data indicate that the α1-antibody can function on multiple levels to affect excitatory/inhibitory balance within neuronal networks of patients with GABA_A_RE. The first appears to operate in real time to alter the functionality of individual receptors, while a second acts on a slower time scale possibly by removing synaptic receptors through antibody-mediated receptor endocytosis. While not examined in detail here, previous studies have explored the role of autoantibodies on receptor internalization. In particular, it has been shown that IgG1-isotype autoantibodies can lead to cross-linking of receptors causing their internalization (Crisp et al., 2019; Dalmau and Graus, 2022). For example, it was shown that NMDAR IgG1-type auto-antibodies promote loss of synaptic receptors in ∼4-h, reaching maximal internalization rates by 12 h (Moscato et al., 2014). Similarly, glycine receptor (GlyR) autoantibodies promoted the endocytosis of receptor in ∼2 h in HEK293 cell and 16 h in motor neurons (Crisp et al., 2019). How much the latter applies to the actions of the α1-antibody is unclear. Our own results revealed a dramatic loss of synaptic receptors following a 24 h antibody incubation, indicating that antibody-mediated endocytosis could contribute to the long-term effects of these antibodies, similar to both GlyR and NMDARs (Kreye et al., 2016; Crisp et al., 2019).

The rapid effects of the α1-antibody, modifying GABA_A_ receptor function within minutes, is not unique and has also been observed for IgG1 autoantibodies targeting inhibitory GlyR and GABA_B_ receptors (Nibber et al., 2017; Dalmau and Graus, 2022). For example, Fab fragments of antibodies targeting the GlyRs could reduce mIPSC frequency in as little as 15 min following its addition to dissociated spinal cord cultures (Crisp et al., 2019). Mechanistically, how might the α1-antibody elicit such rapid changes in GABA_A_R function? One possibility is that it triggers a conformational change that promotes rapid receptor internalization. Alternatively, it could act on GABA binding sites, as a competitive antagonist, similarly to gabazine (Johnston, 2013; Ghit et al., 2021). Ultrastructural epitope mapping of the α1-antibody binding site and initial electrophysiological recording from transfected cells support this concept (Noviello et al., 2022), yet more detailed biophysical studies are needed to define the actual molecular mechanism.

### α1γ2-antibody, an unresolved puzzle

Initial studies on the α1γ2-antibody suggested that it could elicit strong neuronal network dysfunction since P21 rats receiving a cerebroventricular infusion showed increases in ictal events similar to the α1-antibody (Kreye et al., 2021). Thus, our initial expectation was that similar to the α1-antibody, the α1γ2-antibody would alter GABA_A_R distribution and/or functional properties. However, although this antibody decorated ∼40% of GABAergic VGAT-positive synapses, there was no significant redistribution of these receptors after a 24-h treatment (Fig. 2). If anything, this antibody accumulated, which may be related to its low affinity (Kreye et al., 2021; Fig. 2*D–F*). The reason why this antibody only decorated 40% of the synapses could be because of the relative low expression of α1γ2-containing receptors in the striatum which is around 50% (Boccalaro et al., 2019). However, low labeling because of technical reasons cannot completely be ruled out. Surprisingly, incubating striatal autaptic neurons for 24 h did not affect total GABA-mediated currents (Fig. 3*F*), evoked IPSC amplitudes (Fig. 3*C*), or mIPSC frequency and amplitude (Fig. 3*K–M*). Furthermore, increasing the concentration of the α1γ2 antibody to 5 μg/ml did not affect spiking frequency of cortical-striatal cultures compared with control-antibody or 1 μg/ml α1γ2 both after a 24- or 1-h incubation (Figs. 4, 6*A*,*B*, respectively). Together these data raise a fundamental question of how such an antibody could increase ictal events when infused into the brains of rats (Kreye et al., 2021), but not cortical-striatal neurons in cultures.

Two major differences between these two preparations exist: (1) the absence of naturally occurring allosteric modulators, such as endozepines or neurosteroids, which normally enhance GABA_A_R function to maintain E/I balance (Olsen, 2015) as well as (2) microglia. Regarding the former, the ability of the α1γ2-antibody to bind both subunits hints that this antibody might occupy the benzodiazepine binding site. However, we failed to detect changes in the response to diazepam, following α1γ2-antibody incubation (Figs. 7, 8). This could be because of a mixture of benzo-sensitive receptor-subtypes on these cells and/or that the α1γ2-antibody blocks the action of other modulators not investigated in this study. Future detailed biophysical studies of single-channel conductance of α1γ2-containing GABA_A_Rs could help resolve this issue.

Although seldom considered as a mechanism associated with autoimmune encephalitis, there is a growing appreciation that microglia and cellular immunity play important roles in the etiology of a number of brain disorders with focal lesions, epilepsy and cognitive decline, including Alzheimer’s disease (Sarlus and Heneka, 2017). Here, it is worth noting that both the α1 and α1γ2-antibodies are of the human IgG1 (hIgG1) subtype with binding sites for both complement and Fc-γ receptors (Bournazos et al., 2017). This raises the possibility that microglia *in situ* could actively strip hIgG1-antibodies/GABA_A_ receptor complexes from the surfaces of neurons, increasing network excitability as a consequence of reduced inhibitory drive, a mechanism worth exploring.

### General directions for autoimmune encephalitis

This study demonstrates that pathogenic autoantibodies can act through many mechanisms within the same disease or sometimes even within the same autoantibody and highlights the complexity underlying GABA_A_R encephalitis and related diseases. It is becoming clear that these mechanisms can range from acute effects on receptors, through long-term receptor loss, to silent binding and possible recruitment of immune system components. This could also mean that each patient has a unique disease signature based on the combination of autoantibodies detected in CSF. This complexity makes it challenging to implement new treatment strategies other than general immunosuppressants; however, further understanding of the type of autoantibodies present in patients and their modes of action could lead to a more individualistic approach to treatment.

## Synthesis

Reviewing Editor: Viji Santhakumar, University of California Riverside Decisions are customarily a result of the Reviewing Editor and the peer reviewers coming together and discussing their recommendations until a consensus is reached. When revisions are invited, a fact-based synthesis statement explaining their decision and outlining what is needed to prepare a revision will be listed below. The following reviewer(s) agreed to reveal their identity: Suchitra Joshi. Note: If this manuscript was transferred from JNeurosci and a decision was made to accept the manuscript without peer review, a brief statement to this effect will instead be what is listed below.

Cellular mechanisms underlying neurological impairment due to autoantibodies are incompletely understood and the treatments that could completely resolve the clinical symptoms are lacking. This study shows that antibodies targeting GABA-A receptors could have distinct effects on neuron’s physiology. While antibodies targeting GABA-A receptors are expected to alter GABA-A receptor currents in the neurons and alter excitability, these studies demonstrating diversity in the nature of changes are necessary to understand the pathophysiology. The experiments are rigorously performed and authors have addressed most prior concerns adequately. The work adds to the repertoire of mechanisms mediating ion-channel mediating autoimmune encephalitis.

Specific comments:

1. The statement that most synapses in the striatum do not contain a1g2 needs to be placed in the context of inherent experimental limitations and modified to be less categorical. Since the study uses a single antibody, there are a host of technical reasons for why there may be an apparent lack of synaptic labeling, without actual absence of the a1g2-containing GABARs themselves.

2. The authors hypothesize in a couple places that striatal synapses contain other subunit combinations. However, a2,a3 or a5bg2 containing synapses are likely to have slower decay kinetics than a1g2. The slow decay of eIPSCs, but not mIPSCs raise the other possibility of redistribution of the GABARs to the peri/extrasynaptic space. The authors may consider discussing this interesting aspect.

## Author Response

Comments:

Computational Neuroscience Model Code Accessibility Comments for Author (Required):

While this is not a computational study. The authors use custom code OpenView software (Dr. Noam Ziv, Technion Institute, Haifa, Israel). The authors need to include a statement on code availability.

We would like to thank the editor for this comment and agree with your policy of transparency and accessibility in science. Therefore, we have added a subheading in the material and methods section specifying how Dr. Noam Ziv and the corresponding code can be assessed (see line 265-268).

1. The statement that most synapses in the striatum do not contain a1g2 needs to be placed in the context of inherent experimental limitations and modified to be less categorical. Since the study uses a single antibody, there are a host of technical reasons for why there may be an apparent lack of synaptic labeling, without actual absence of the a1g2-containing GABARs themselves.

We agree with the reviewer that more nuance might be necessary in regards to this conclusion. Our α1γ2-antibody only binds to ∼40% of synapses in our immunocytochemical staining. While a study showed that synapses in the striatum only contain α1γ2-receptors in 40-50% of the synapses (Boccalaro et al. J Comp Neurol 2019), this does not rule out that the low labeling percentage could be due to other technical factors. We have added a sentence to the manuscript to address this limitation (see line 637-641)

2. The authors hypothesize in a couple places that striatal synapses contain other subunit combinations. However, a2,a3 or a5bg2 containing synapses are likely to have slower decay kinetics than a1g2. The slow decay of eIPSCs, but not mIPSCs raise the other possibility of redistribution of the GABARs to the peri/extrasynaptic space. The authors may consider discussing this interesting aspect.

We very much agree with this excellent suggestion. Although we have hinted to this idea in several places in the manuscript, we have addressed it more explicitly in the discussion. (see line 580-584.
