# Supplementary figures and images for: Differential Modes of Action of α1- and α1γ2-Autoantibodies Derived from Patients with GABAAR Encephalitis
Source: eNeuro. 2022 Dec 9;9(6):ENEURO.0369-22.2022. doi: 10.1523/ENEURO.0369-22.2022 (PMC9765394; doi:10.1523/ENEURO.0369-22.2022)

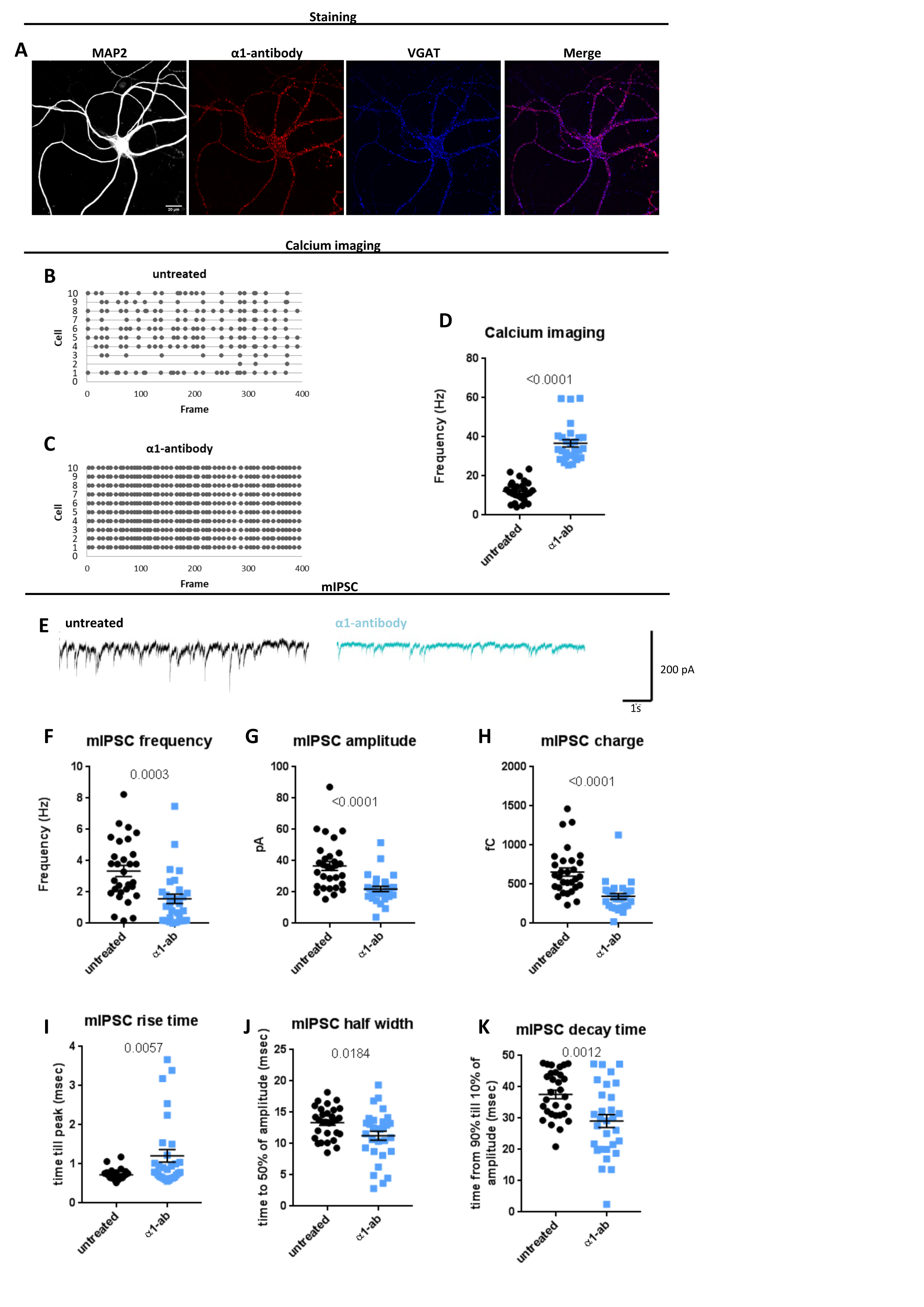

Supplement: Extended Data Figure 6-1 — Effects of α1-antibody 6 h after antibody washout. Example of neurons stained for MAP2 (white), α1-antibody (red), VGAT (blue), and Merge (α1-antibody and VGAT) 6 h after antibody washout (A). Example spike plots of untreated (B) and α1-antibody-treated (C) cortical-striatal cultures during calcium imaging experiments. D, Spiking frequency is still increased 6 h after α1-antibody washout t(52) = 11.51, p < 0.0001, t test, untreated 12.24 ± 0.98 Hz, α1-antibody 36.71 ± 1.88 Hz. E, Example mIPSC traces. Even 6 h after α1-antibody removal, we see decreases in frequency t(58) = 3.838, p = 0.0003, t test, untreated 3.35 ± 0.36 Hz, α1-antibody 1.56 ± 0.30 Hz, (F) amplitude t(46.71) = 4.44, p < 0.0001, Welch’s t test, untreated 36.65 ± 2.86 pA, α1-antibody 21.93 ± 1.68 pA, (G) charge t(57) = 4.77, p < 0.0001, t test, untreated 655.8 ± 53.81 fC, α1-antibody 345.6 ± 35.69 fC, (H) half-width t(58) = 2.426, p = 0.0184, t test, untreated 13.35 ± 0.46 ms, α1-antibody 11.26 ± 0.73 ms, (J) and decay time t(58) = 3.414, p = 0.0012, t test, untreated 37.57 ± 1.38 ms, α1-antibody 29.07 ± 2.07 ms (K), and an increase in rise time t(30.38) = 2.976, p = 0.0057, Welch’s t test, untreated 0.72 ± 0.03 ms, α1-antibody 1.2 ± 0.16 ms (I). Download Figure 6-1, TIF file. [file enu-eN-NWR-0369-22-s02.tif]
